# TDP43 cytoplasmic mislocalization initiates mitochondrial dysfunction and intercellular senescence propagation in intervertebral disc degeneration

**DOI:** 10.1038/s12276-026-01709-z

**Published:** 2026-05-01

**Authors:** Zhiwei Liao, Dingchao Zhu, Zixuan Ou, Mingke Zhao, Kangcheng Zhao, Bide Tong, Cao Yang, Shuai Li

**Affiliations:** Department of Orthopaedics, Union Hospital, Tongji Medical College, Huazhong University of Science and Technology, Wuhan, China

**Keywords:** Molecular biology, Senescence

## Abstract

Intervertebral disc degeneration (IDD), a leading cause of low back pain, involves progressive dysfunction of nucleus pulposus (NP) cells and extracellular matrix degradation. The pathological mechanisms underlying IDD remain complex and lack comprehensive elucidation. This study identifies the RNA-binding protein TDP43 as a central driver of IDD pathogenesis through analysis of human clinical specimens and rodent models. We demonstrate that TDP43 expression escalates proportionally with disc degeneration severity and aberrantly accumulates in the mitochondria of degenerative NP cells. This mitochondrial mislocalization triggers nuclear pore complex impairment, mitochondrial membrane potential collapse, and irreversible cellular senescence. Critically, TDP43 is secreted within mitochondrial-derived vesicles, which function as intercellular mediators that propagate pro-inflammatory cytokines and senescence phenotypes to neighboring NP cells. Both genetic and pharmacological inhibition of vesicular TDP43 effectively attenuated mitochondrial dysfunction and reduced cellular senescence and ultimately decelerated IDD progression in vivo and in vitro. Our findings establish TDP43-loaded mitochondrial-derived vesicles as novel mediators of intercellular pathology and nominate TDP43 as a therapeutic target for IDD intervention.

## TDP43 drives intervertebral disc degeneration through mitochondria

Intervertebral disc degeneration (IDD) is a leading cause of chronic low back pain, driven by complex pathophysiological mechanisms. This study identifies TDP43, an RNA-binding protein, as a key player in IDD, showing its elevated expression in degenerated nucleus pulposus cells and its correlation with disc degeneration severity. Through transcriptomic analysis, the authors reveal that TDP43 localizes to mitochondria, contributing to mitochondrial dysfunction and cellular senescence. The study demonstrates that nucleus pulposus cells secrete TDP43-enriched mitochondrial-derived vesicles, which propagate senescence and inflammation in recipient cells. Inhibition of TDP43 in these vesicles alleviates cellular senescence and slows IDD progression in vitro and in vivo. These findings highlight TDP43 as a potential therapeutic target for IDD, suggesting that future research should explore the molecular mechanisms of the role of TDP43 in mitochondrial dysfunction and its potential as a treatment avenue. This summary was initially drafted using artificial intelligence, then revised and fact-checked by the author.

## Introduction

Intervertebral disc degeneration (IDD) represents one of the primary causes of chronic low back pain, characterized by multifaceted pathophysiological mechanisms^1^. The intervertebral disc comprises the nucleus pulposus (NP), annulus fibrosus (AF), and cartilaginous endplates, with the NP having a pivotal role in maintaining biomechanical homeostasis^2^. Stressors including diminished nutrient supply, hypoxia, lactate accumulation, and sustained mechanical loading contribute to NP cell injury^3–5^. Progressive NP cell dysfunction and degradation of the extracellular matrix (ECM) ultimately lead to loss of disc elasticity and structural integrity^6^. Notably, degenerated discs exhibit increased senescent and fibrotic NP cells^7^, which secrete pro-inflammatory cytokines that exacerbate ECM catabolism^3^. Among these, mitochondrial dysfunction is closely associated with cellular inflammation and represents one of the key pathological mechanisms in the initiation and progression of IDD^8,9^. However, the pathology of IDD is complex and influenced by various intrinsic and extrinsic factors. Comparative analysis between normal and degenerated NP cells may elucidate molecular pathways underlying IDD progression and identify therapeutic targets.

In recent years, a growing body of research has demonstrated that RNA-binding proteins (RBPs) have crucial roles in disease pathogenesis^10,11^. RBPs interact with protein-coding genes, messenger RNAs, and non-coding RNAs to exert diverse biological functions, including regulation of mRNA stability and modulation of non-coding RNA synthesis^12^. Emerging evidence indicates that diverse RBPs are involved in the regulation of NP function and the progression of IDD^13,14^. These RBPs influence critical biological processes such as ECM metabolism, inflammatory responses, cellular senescence, apoptosis, and ferroptosis, thereby participating in the molecular regulation of IDD pathogenesis^13^. Identification of key RBPs that regulate disc degeneration could facilitate the discovery of novel therapeutic targets for molecular interventions in IDD.

TDP43 (TAR DNA-binding protein 43), a ubiquitously expressed nuclear RBP, primarily participates in post-transcriptional regulation, RNA splicing, and RNA transport^15^. Recent studies have revealed that aberrant aggregation and dysfunction of TDP43 are closely associated with neurodegenerative disorders, including amyotrophic lateral sclerosis, frontotemporal dementia, and Alzheimer disease^15,16^. TDP43 modulates inflammatory responses through regulating the stability of various inflammatory mediators, whereas its pathological aggregation disrupts normal cellular metabolic processes, leading to cellular dysfunction and death^17^. Research has demonstrated altered subcellular localization of TDP43 under pathological conditions, with cytoplasmic translocation resulting in functional impairment of cellular organelles through accumulated TDP43 (refs. ^18,19^). Nevertheless, to our knowledge, the precise role and molecular mechanisms of TDP43 in IDD remain to be elucidated.

This study identifies TDP43 as a differentially expressed RBP in degenerated and stressed NP cells through comparative transcriptomic analysis. It demonstrates that elevated TDP43 expression correlating with disc degeneration grades in both clinical specimens and animal models. Mitochondrial localization of TDP43 associates with mitochondrial dysfunction and cellular senescence in degenerated NP cells. Mitochondrial-derived vesicles (MDVs), as a selective mechanism for removing mitochondrial components, have an important role in intercellular communication and cellular homeostasis, influencing both the originating and surrounding cells. We aim to investigate whether MDVs have a role in the inflammation and senescence of NP cells. Mechanistically, NP cells secrete TDP43-enriched MDVs that propagate senescence phenotypes and amplify inflammatory responses in recipient cells. Genetic and pharmacological inhibition of TDP43 in vesicles alleviates cellular senescence and decelerates IDD progression in vitro and in vivo. Our findings reveal the pathological role of TDP43 and propose TDP43 as a novel therapeutic target for IDD intervention.

## Materials and methods

### Tissue samples

All involved experiments were conducted according to the ethical policies and procedures approved by the Ethics Committee of Tongji Medical College, Huazhong University of Science and Technology. Human NP specimens were obtained during discectomy procedures performed as part of spinal fusion surgery. Collection strictly adhered to the Declaration of Helsinki, with preoperative written informed consent from all patients. Preoperative MRI scans (*n* = 12 per grade) classified disc degeneration severity using the Pfirrmann grading system (grades I–V), characterized radiographically as follows: grade I: homogeneous hyperintensity (bright white), indicating intact hydration; grade II: discernible NP/AF boundary, despite emerging inhomogeneity; grade III: grayish NP signal intensity with complete loss of NP/AF demarcation; grade IV: marked hypointensity (dark gray/black) with initial disc space narrowing; grade V: structural disintegration featuring obliterated disc space and indistinct NP/AF architecture. All specimens were immediately preserved via immersion in 4% paraformaldehyde or cryopreservation in liquid nitrogen for subsequent analysis.

### Cell culture

Human NP cells were isolated from surgically resected NP specimens through enzymatic dissociation. Primary cells were maintained in complete culture medium consisting of DMEM/F-12 supplemented with 15% fetal bovine serum (Cell-Box, China) under standard culture conditions (37 °C, 5% CO_2_). The isolation protocol comprised sequential steps: first, specimens underwent triple PBS washing cycles to remove blood contaminants; second, tissue fragmentation using sterile surgical scissors; third, incubation with 0.25% type II collagenase under sterile conditions at 37 °C for 4 h and sequential PBS washes (×2) followed by centrifugation at 250 × *g* for 5 min to pellet digested cells. Finally, cells were resuspended in fresh DMEM/F-12 medium and underwent medium renewal every 72 h. Experimental procedures utilized second-passage (P2) cells to minimize phenotypic drift associated with prolonged in vitro expansion.

### Statistical analysis

Data analysis was performed using GraphPad Prism 8 (La Jolla, CA, USA), with results expressed as mean ± standard deviation (SD). Intergroup differences were analyzed using two-tailed unpaired *t* tests for pairwise comparisons. Multigroup analyses used one-way or two-way analysis of variance with Tukey’s post hoc test. Statistical significance thresholds were set at the 0.05 level (*P < 0.05, **P < 0.01, ***P < 0.001, and ^ns^*P* ≥ 0.05).

### Supplementary materials

Detailed methodological descriptions, including experimental materials and data analysis procedures, are provided in the Supplementary materials section. Information for antibodies and reagents was provided in Supplementary Table 1. Information for primers of genes was provided in Supplementary Table 2.

## Results

### Validation of TDP43 protein dysregulation and subcellular localization in NP cells

**Fig. 1:**
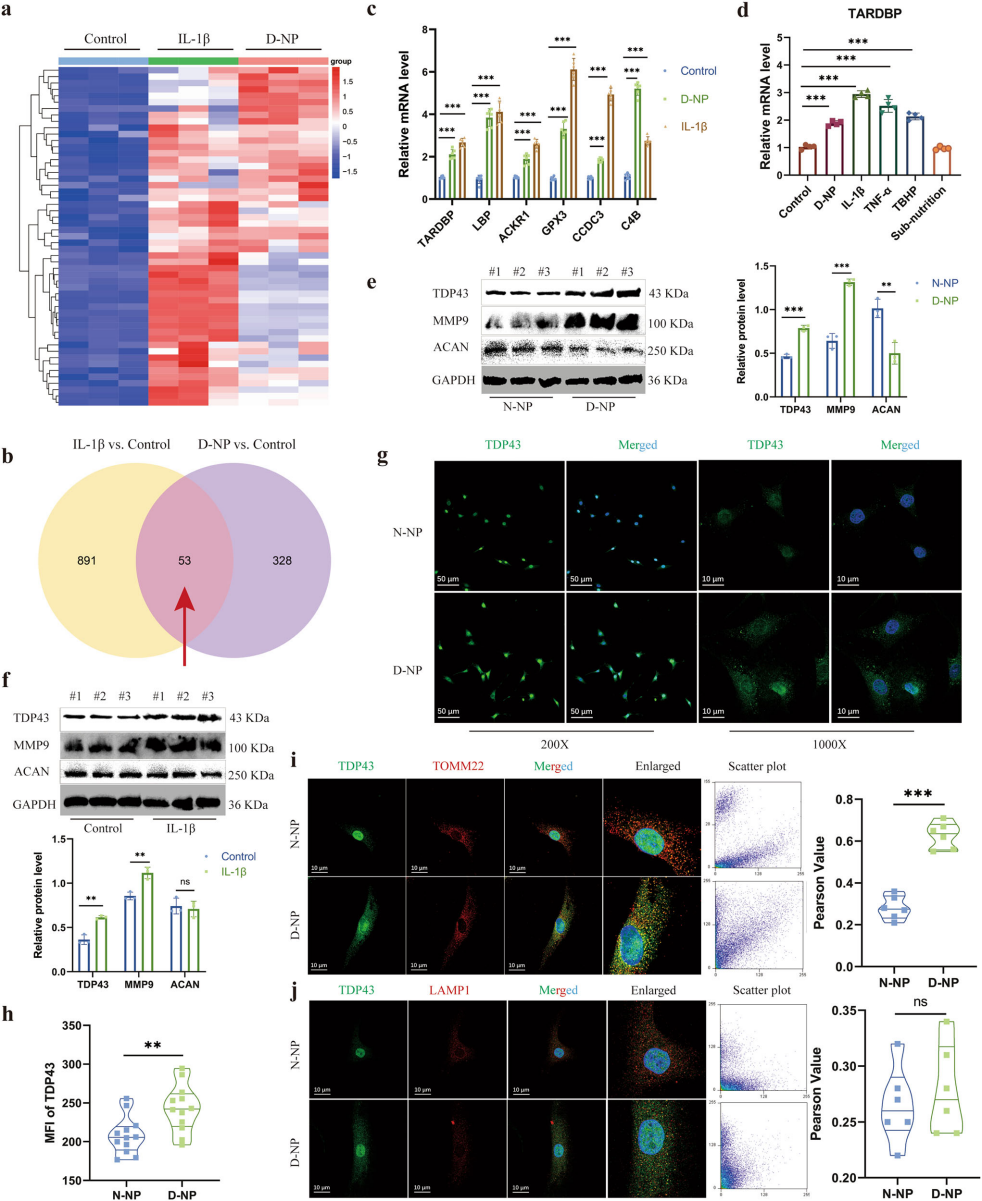
TDP43 expression increased in stressed and degenerative NP cells. **a** Heatmap of differentially expressed genes in nucleus pulposus (NP) cells (control versus IL-1β and degenerative groups). **b** Venn diagram of upregulated genes both in IL-1β and degenerative groups. **c** Relative mRNA levels of selected genes in NP cells in respective groups. **d** Relative mRNA levels of TARDBP in diverse NP cell models. **e** Western blot and relative protein levels of TDP43, MMP9, and ACAN in normal and degenerative (N-NP and D-NP, respectively) NP cells. **f** Western blot and relative protein levels of TDP43, MMP9, and ACAN in IL-1β-treated and control NP cells. **g**,**h** Immunofluorescence images and mean fluorescence intensity of TDP43 in N-NP and D-NP groups. **i** Immunofluorescence images, scatter plot, and Pearson value of TDP43 and TOMM22 colocalization in N-NP and D-NP groups. **j** Immunofluorescence images, scatter plot and Pearson value of TDP43 and LAMP1 colocalization in N-NP and D-NP groups. Data are shown as mean ± SD (*n* = 3 independent experiments). **P < 0.01, ***P < 0.001, ns (not significant) by Student’s *t* test or one-way analysis of variance. MFI, mean fluorescence intensity; MMP, matrix metalloproteinase.

To identify pivotal molecules involved in IDD, we performed transcriptome analysis of NP cells under inflammatory stimuli (IL-1β treatment) and degenerative conditions (Fig. 1a). Compared with controls, the IL-1β-treated group exhibited 944 upregulated differentially expressed genes, whereas the degeneration group showed 381 upregulated differentially expressed genes, with 53 genes concurrently upregulated in both IL-1β-treated and degenerative NP cells (Fig. 1b). Functional annotation of the 53 genes via GO and KEGG analyses (Supplementary Fig. 1a,b) identified 6 genes related to “cell inflammation” and “stress response”. These 6 genes demonstrated consistent upregulation in IL-1β-treated and degenerative NP cells (Fig. 1c), among which TARDBP displayed strong associations with degenerative pathologies. Although IDD is a prevalent musculoskeletal degenerative disorder, the role of TARDBP in this context remains undefined. We established multiple NP cell injury/stress models, including IL-1β, tumor necrosis factor (TNF)-α, tert-butyl hydroperoxide, and nutrient deprivation, and assessed the expression of TARDBP. TARDBP was markedly elevated across all cell models (Fig. 1d), suggesting its significance in NP cell degeneration.

Protein analysis revealed increased TDP43 (encoded by TARDBP) expression in degenerative NP cells compared with normal controls, coupled with catabolic metabolism of ECM (Fig. 1e). In IL-1β-induced inflammatory models, TDP43 upregulation coincided with elevated expression of MMP9 (Fig. 1f). Immunofluorescence confirmed the increased TDP43 expression in degenerative NP cells accompanied by prominent cytoplasmic distribution (Fig. 1g,h). Further subcellular localization studies using organelle markers demonstrated significant TDP43 colocalization with mitochondria (labeled by TOMM22) in degenerative NP cells, whereas no obvious colocalization with lysosomes (LAMP1-positive) was observed (Fig. 1i,j). This indicates pathophysiological accumulation of TDP43 on mitochondria in NP cells, although its functional impact on mitochondrial function remains unknown. Collectively, elevated TDP43 expression across NP cell injury models suggests its involvement in the degenerative cascade of IDD.

### Correlation of TDP43 with disc degeneration severity in human samples and rat models

**Fig. 2:**
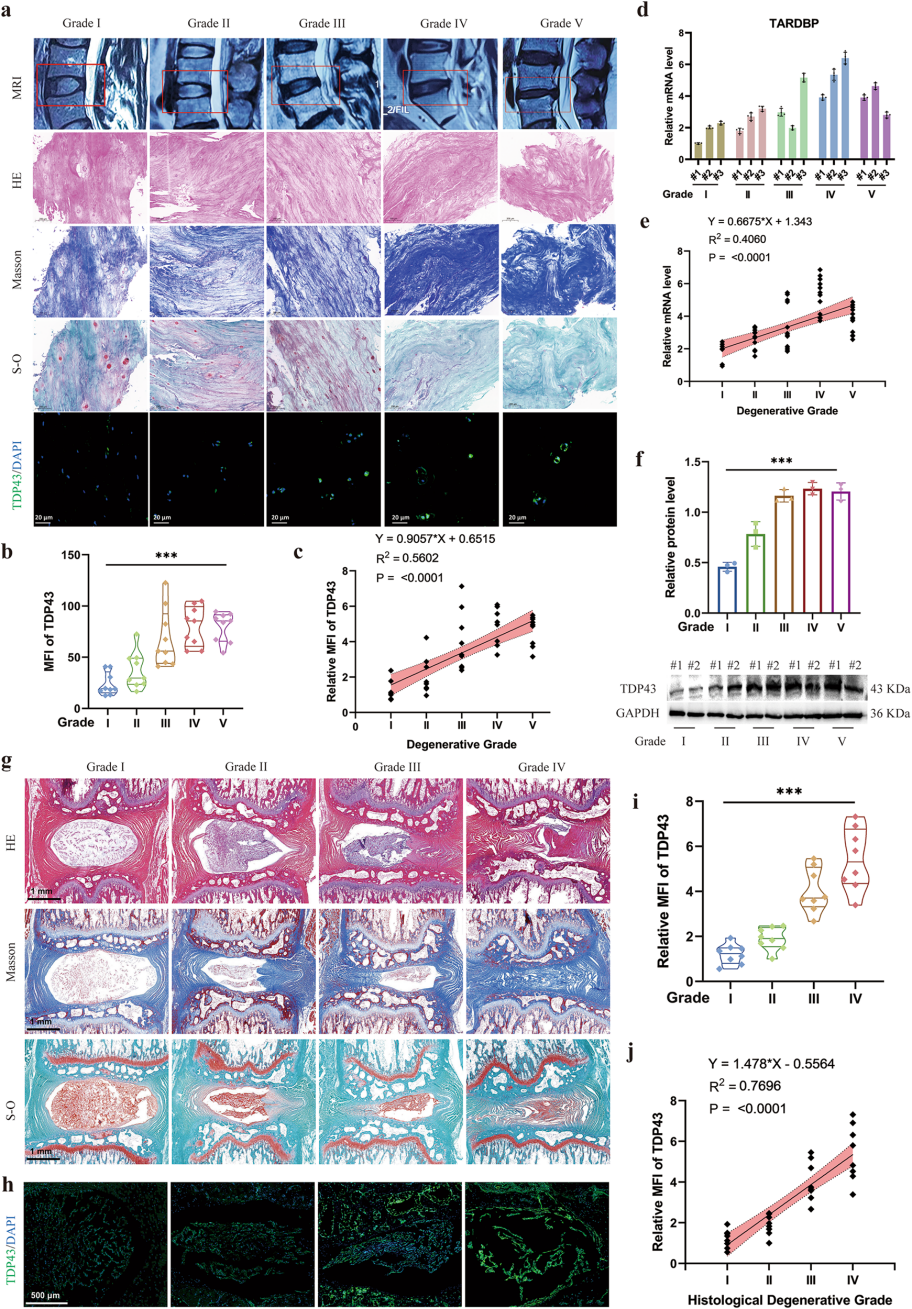
TDP43 expression increased in human and rat degenerate disc tissues. **a** Representative MRI images, hematoxylin–eosin (HE), Masson, and Safranin O-fast green (S-O) staining, TDP43 immunofluorescence of intervertebral disc samples with different degenerative grades (grades I–V). **b** Mean fluorescence intensity (MFI) of TDP43 in disc tissues. **c** Correlation analysis between the MRI grade and the relative MFI of TDP43. **d** Relative mRNA level of TARDBP gene in disc tissues. **e** Correlation analysis between the MRI grade and the mRNA level of TARDBP. **f** Western blot and relative protein levels of TDP43 in disc tissues. **g** Representative HE, Masson, and S-O staining of rat disc with different degenerative grades (grades I–IV). **h**,**i**, Immunofluorescence images and MFI of TDP43 in rat disc. **j** Correlation analysis between the histological degenerative grade and the relative MFI of TDP43. Data are shown as mean ± SD (*n* = 3 independent experiments). ***P < 0.001 by one-way analysis of variance, or linear regression. DAPI, 4′,6-diamidino-2-phenylindole.

To delineate TDP43 expression during IDD progression, human disc specimens were stratified into degeneration grades using the Pfirrmann classification based on T2-weighted MRI. Histological analysis revealed progressive decline in cellularity, loss of ECM, and enhanced fibrosis across ascending degeneration grades (Fig. 2a). Immunofluorescence demonstrated MRI grade-dependent upregulation of TDP43 (Fig. 2b). Regression analysis confirmed a significant positive correlation between mean fluorescence intensity (MFI) of TDP43 and degeneration grade (*R*^2^ = 0.5602, *P* < 0.0001; Fig. 2c). Parallel transcript quantification demonstrated concordant elevation of TARDBP mRNA levels (Fig. 2d), which significantly correlated with degeneration severity (*R*^2^ = 0.406, *P* < 0.0001; Fig. 2e). Western blotting further validated the significantly increased expression of TDP43 protein in high-grade degenerative discs (Fig. 2f).

Rat IDD model was conducted via tail disc puncture and graded by histopathological evaluation of NP, AF, and cartilage endplate （CEP） tissues. High-grade degeneration exhibited NP degradation replaced by fibrotic tissue, disrupted annular lamellae with collagen fragmentation, and CEP degeneration with significant disc height reduction (Fig. 2g). Immunofluorescence confirmed the significant TDP43 elevation in NP area with higher degeneration grade (Fig. 2h,i). Regression analysis demonstrated a robust positive correlation between the MFI of TDP43 and degeneration grade (*R*^2^ = 0.7696, *P* < 0.0001; Fig. 2j). These results demonstrated that TDP43 expression increases with degeneration severity in both human and rat IDD models, showing a consistent positive correlation with histopathological grading.

### Pathogenic role and therapeutic intervention of TDP43 in rat disc degeneration

**Fig. 3:**
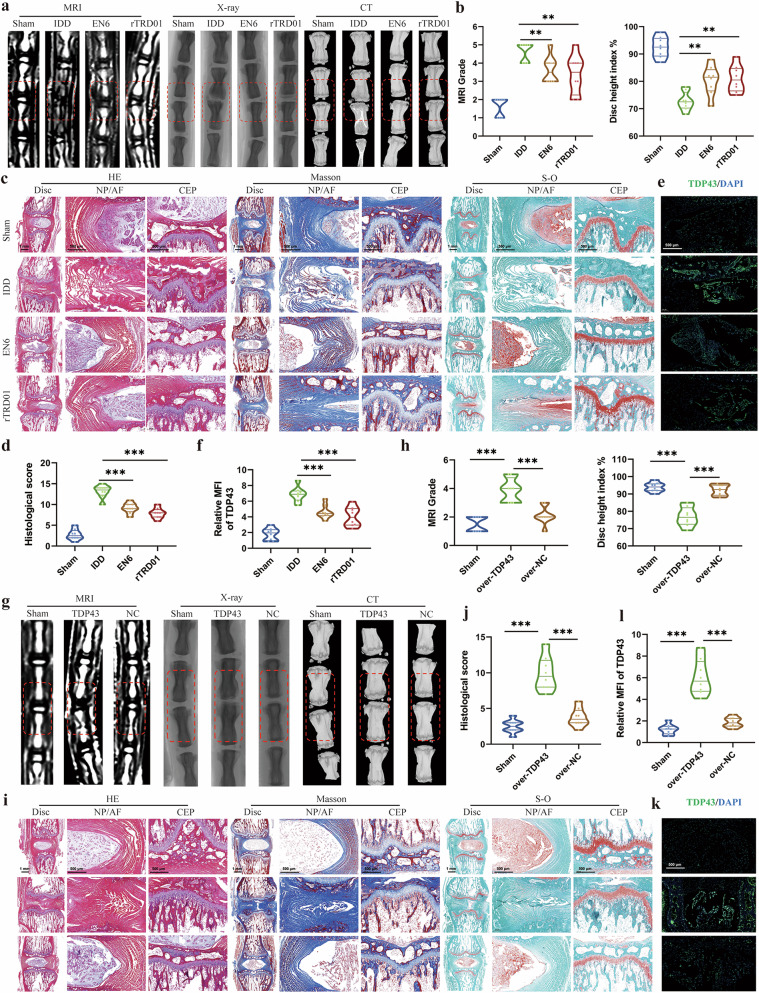
TDP43 intervention affects the progression of rat disc degeneration. **a** Representative MRI, X-ray, and CT images of rat discs injected with or without EN6 or rTRD01. **b** MRI grade and disc height index variation of rat discs in respective groups. **c** Representative MRI images, hematoxylin–eosin (HE), Masson, and S-O staining of rat discs in respective groups. **d** Histological scores of rat discs in respective groups. **e**,**f** Immunofluorescence images and relative mean fluorescence intensity (MFI) of TDP43 in respective groups. **g**, Representative MRI, X-ray, and CT images of rat discs injected with TDP43 plasmid or normal control (NC) plasmid. **h** MRI grade and disc height index variation of rat discs in respective groups. **i** Representative MRI images, HE, Masson, and S-O staining of rat discs in respective groups. **j** Histological scores of rat discs in respective groups. **k**,**l** Immunofluorescence images and relative MFI of TDP43 in respective groups. Data are shown as mean ± SD (*n* = 3 independent experiments). **P < 0.01 and ***P < 0.001 by one-way analysis of variance. AF, annulus fibrosus; IDD, intervertebral disc degeneration; NP, nucleus pulposus.

To investigate the therapeutic potential of TDP43 modulation in IDD, we administered specific TDP43 inhibitors (EN6 and rTDR01) in a rat caudal disc puncture model. Puncture-induced degenerative discs exhibited characteristic MRI findings including reduced hydration (evidenced by decreased T2 signal intensity), elevated MRI degeneration grades, and diminished disc height index (DHI) on X-ray/CT assessment (Fig. 3a,b). Following intradiscal delivery of TDP43 inhibitors, quantitative imaging analysis demonstrated significant therapeutic effects: MRI degeneration grades were markedly reduced whereas DHI values increased substantially compared with sham controls. Histological evaluation further confirmed aggravated degeneration in the IDD group, showing NP depletion, annular fibrosus ruptures, and CEP degeneration. Inhibitor-treated groups conversely exhibited mitigated tissue damage with significantly lower degeneration scores (Fig. 3c,d). Immunofluorescence analysis validated decreased TDP43 fluorescence intensity within discs treated with TDP43 inhibitors (Fig. 3e,f), functionally linking TDP43 suppression to attenuated IDD progression.

Subsequently, we performed transfection of TDP43 plasmid into the rat discs and assessed the degree of disc degeneration. The TDP43-overexpressing group developed elevated MRI degeneration grades and reduced DHI measurements compared with empty vector controls (Fig. 3g,h). Histological examination revealed profound structural deterioration: fibrotic replacement of NP tissue, loss of demarcation between NP and AF, and CEP sclerosis with osteophyte formation (Fig. 3i). Quantitative histological scoring confirmed significantly higher degeneration in the TDP43-overexpressing group (Fig. 3j), which corresponded with elevated TDP43 fluorescence intensity in immunofluorescence (Fig. 3k,l). Taken together, these findings establish TDP43 as a central pathogenic mediator in IDD, wherein its inhibition attenuates degenerative processes, underscoring its potential as a therapeutic target for disc degeneration.

### Nuclear export and mitochondrial localization of TDP43 during NP cell degeneration

**Fig. 4:**
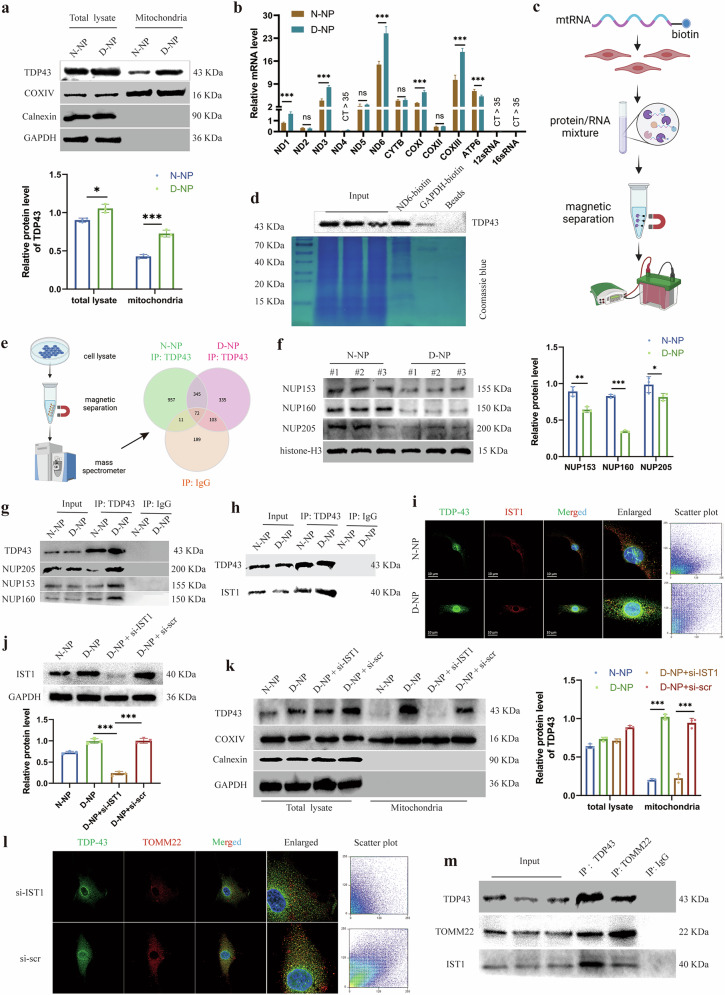
TDP43 tends to form mitochondrial localization in degenerative NP cells. **a** Western blot and relative protein levels of TDP43, COXIV, and Calnexin in total lysate and mitochondrial fractions in normal nucleus pulposus (N-NP) and degenerative NP (NP) (D-NP) cells. **b** Relative mRNA levels of mitochondrial RNA in TDP43 immunoprecipitates. **c** Workflow of biotin-labeled mitochondrial RNA pulldown assay. **d** Western blot and Coomassie blue staining of TDP43 in ND6-biotin pulldown assay. GAPDH-biotin as a negative control and beads as an empty control. **e** Workflow of TDP43 immunoprecipitation in NP cells. Venn diagram of TDP43-binding proteins in D-NP versus N-NP cells. IgG as a negative control. **f** Western blot and relative protein levels of NUP153, NUP160, and NUP205 in nuclear fractions. **g** Immunoprecipitation and immunoblotting of TDP43 and NUP proteins. **h** Immunoprecipitation and immunoblotting of TDP43 and IST1. **i** Immunofluorescence images and scatter plot of TDP43 and IST1. **j** Western blot and relative protein levels of IST1 in NP cells transfected with si-IST1 or si-scr. **k** Western blot and relative protein levels of TDP43, COXIV, and Calnexin in total lysate and mitochondrial fractions in si-IST1 or si-scr transfected NP cells. **l** Immunofluorescence images and scatter plot of TDP43 and IST1 in si-IST1 or si-scr transfected NP cells. **m** Immunoprecipitation and immunoblotting of TDP43, TOMM22, and IST1. Data are shown as mean ± SD (*n* = 3 independent experiments). *P < 0.05, **P < 0.01, ***P < 0.001, ns (not significant) by Student’s *t* test, or one-way analysis of variance.

Building upon prior findings of elevated TDP43 expression with enhanced mitochondrial localization in degenerated NP cells, we isolated mitochondrial fractions and quantified mitochondrial TDP43 content. Immunoblotting revealed significantly increased TDP43 accumulation within mitochondria of degenerated NP cells (Fig. 4a), indicating pathological mitochondrial partitioning. As an RBP, TDP43 potentially associates with mitochondrial transcripts to exert regulatory functions. RNA immunoprecipitation assay against TDP43 demonstrated its binding to multiple mitochondrial RNAs, including ND1, ND3, and ND6 transcripts, with selectively enhanced interactions in degenerated NP cells (Fig. 4b). Subsequent biotin-labeled RNA pulldown using ND6 probes further confirmed direct TDP43-mitochondrial RNA binding (Fig. 4c,d), suggesting that mitochondrial TDP43 accumulation likely impairs mitochondrial nucleic acid metabolism.

Although TDP43 predominantly localizes to nuclei under physiological conditions, the mechanism underlying its mitochondrial translocation remained undefined. Comparative proteomic analysis of TDP43-binding proteins identified 335 uniquely bound proteins in degenerated versus normal NP cells (Fig. 4e). Among these, multiple nucleoporins, including NUP153, NUP160, and NUP205, showed reduced nuclear abundance in degenerated NP cells (Fig. 4f). Co-immunoprecipitation (Co-IP) conversely demonstrated strengthened interactions between TDP43 and these nucleoporins in degenerated NP cell (Fig. 4g). These findings imply that TDP43 may sequester nucleoporins to disrupt nuclear pore complex integrity, thereby promoting its cytosolic mislocalization. Further screening revealed TDP43 interaction with the endosomal sorting complex required for transport-associated protein IST1 (also termed CHMP8). Co-IP assays confirmed constitutive TDP43-IST1 binding (Fig. 4h), with immunofluorescence showing intensified colocalization in degenerative cells (Fig. 4i). To functionally characterize IST1, we designed small interfering RNAs (siRNAs) targeting IST1 and selected the most efficient siRNA (Supplementary Fig. 2a). Transfection with IST1-siRNA significantly suppressed IST1 protein expression (Fig. 4j). Notably, IST1 knockdown did not alter total cellular TDP43 levels but markedly reduced mitochondrial TDP43 content (Fig. 4k), accompanied by diminished TDP43 colocalization with TOMM22 (Fig. 4l). Co-IP assays subsequently established the complex formation among IST1, TDP43, and TOMM22 (Fig. 4m), with immunofluorescence confirming their spatial colocalization (Supplementary Fig. 2b). Collectively, these findings indicate that IST1 facilitates the mitochondrial transport of TDP43, thereby establishing a mechanistic connection between TDP43 and mitochondrial dysfunction during IDD.

### Functional analysis of TDP43 in mitochondrial dysfunction and cellular senescence

**Fig. 5:**
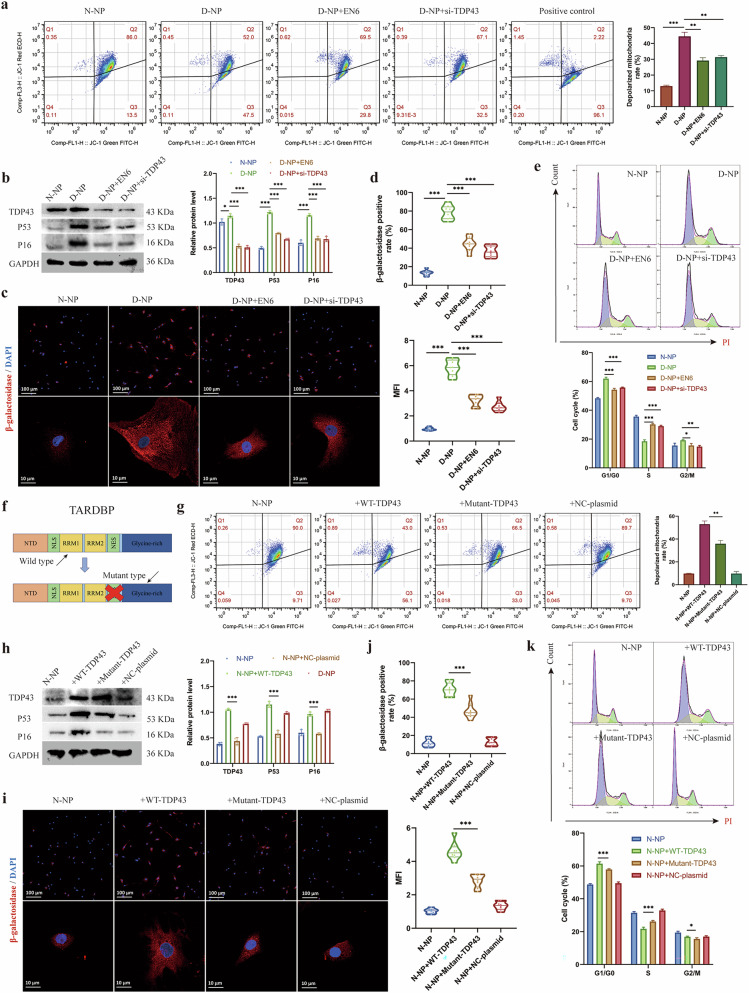
TDP43 induces mitochondrial dysfunction and cellular senescence in NP cells. **a** Flow cytometry of JC-1 staining and depolarized mitochondria rate of nucleus pulposus (NP) cells treated with EN6 or transfected with si-TDP43. **b** Western blot and relative protein levels of TDP43, P53, and P16 in respective groups. **c**,**d** Immunofluorescence images, positive cell rate, and mean fluorescence intensity (MFI) of β-galactosidase in respective groups. **e** Flow cytometry of PI staining and cell cycle modeling in respective groups. **f** Diagram of mutant and wild-type TDP43. **g** Flow cytometry of JC-1 staining and depolarized mitochondria rate of NP cells transfected with mutant or wild-type TDP43 plasmids. **h** Western blot and relative protein levels of TDP43, P53, and P16 in respective groups. **i**,**j** Immunofluorescence images, positive cell rate, and MFI of β-galactosidase in respective groups. **k** Flow cytometry of PI staining and cell cycle modeling in respective groups. Data are shown as mean ± SD (*n* = 3 independent experiments). *P < 0.05, **P < 0.01, ***P < 0.001 by one-way analysis of variance. DAPI, 4′,6-diamidino-2-phenylindole; D-NP, degenerative nucleus pulposus; N-NP, normal nucleus pulposus.

To establish the pathogenic contribution of TDP43 in IDD, we assessed mitochondrial functionality in NP cells. Degenerated cells displayed markedly reduced mitochondrial membrane potential (MMP) coupled with increased depolarized mitochondria. Intervention with TDP43 inhibitors or siRNA restored MMP and substantially suppressed mitochondrial depolarization (Fig. 5a). Concurrent attenuation of senescence markers was observed, as evidenced by reduced protein expression of P53 and P16 in TDP43-knockdown groups (Fig. 5b). Immunofluorescence quantification confirmed elevated proportions of β-galactosidase-positive cells and enhanced MFI in degenerated NP cells, both of which were significantly reversed upon TDP43 suppression (Fig. 5c,d). Flow cytometry further demonstrated ameliorated cell cycle arrest, characterized by decreased G1/G0-phase and increased S-phase populations following TDP43 interference (Fig. 5e).

Besides, TDP43 overexpression in healthy NP cells impaired MMP and expanded depolarized mitochondrial fractions (Supplementary Fig. 3a), while elevating P53 and P16 expression (Supplementary Fig. 3b). SA-β-gal activity (positive cell ratio and MFI) was robustly augmented in TDP43-overexpressing groups (Supplementary Fig. 3c,d), with concomitant cell cycle prolongation reflected by increased G1/G0-phase and reduced S-phase populations (Supplementary Fig. 3e). In addition, we constructed a mutant TARDBP plasmid lacking the NES sequence responsible for nuclear export (Fig. 5f). Compared with wild type, mutant TDP43-expressing cells exhibited restored MMP with attenuated mitochondrial depolarization (Fig. 5g). It also revealed that the downregulated P53/P16 expression (Fig. 5h) diminished SA-β-gal positivity and MFI (Fig. 5i,j) and reduced G1/G0-phase and elevated S-phase cell rates (Fig. 5k) in mutant TDP43-expressing NP cells. In summary, these results demonstrate that cytosolic mislocalization of TDP43 instigates mitochondrial dysfunction, accelerates cellular senescence, and disrupts cell cycle in NP cells.

### TDP43 transportation and encapsulation into mitochondrial-derived vesicles in NP cells

**Fig. 6:**
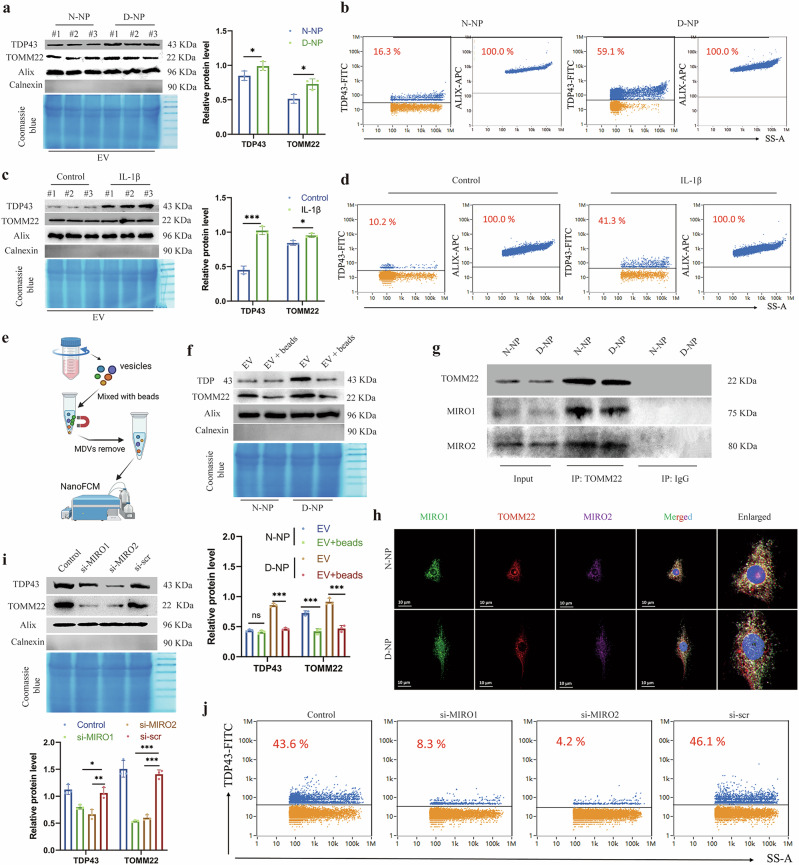
Mitochondrial TDP43 is loaded into mitochondrial-derived vesicles. **a** Western blot and relative protein levels of TDP43, TOMM22, and Alix in extracellular vesicle (EV) fractions from N-NP and D-NP cells. **b** Nanoscale flow cytometry (NanoFCM) analysis of TDP43-FITC in EV fractions from N-NP and D-NP cells. **c**, Western blot and relative protein levels of TDP43, TOMM22, and Alix in EV fractions from IL-1β-treated and control cells. **d** NanoFCM analysis of TDP43-FITC in EV fractions from IL-1β-treated and control cells. **e** Workflow of mitochondrial-derived vesicles (MDVs) removed with immunomagnetic TOMM22 beads. **f** Western blot and relative protein levels of TDP43, TOMM22, and Alix in EV fractions treated with or without TOMM22 beads. **g** Immunoprecipitation of TOMM22, MIRO1, and MIRO2 in N-NP and D-NP cells. **h** Immunofluorescence images of TOMM22, MIRO1, and MIRO2 in N-NP and D-NP cells. **i**, Western blot and relative protein levels of TDP43, TOMM22, and Alix in EV fractions from si-MIRO1-transfected or si-MIRO2-transfected NP cells. **j**, NanoFCM analysis of TDP43-FITC in EV fractions from si-MIRO1-transfected or si-MIRO2-transfected NP cells. Data are shown as mean ± SD (*n* = 3 independent experiments, *n* = 4 independent cell isolations for NanoFCM). *P < 0.05, **P < 0.01, ***P < 0.001 by Student’s *t* test, one-way or two-way analysis of variance. D-NP, degenerative nucleus pulposus; FITC, fluorescein isothiocyanate; N-NP, normal nucleus pulposus.

Building on our findings that pathological TDP43 accumulates within mitochondria to disrupt bioenergetics, we investigated whether mitochondrial quality control mechanisms regulate mitochondrial TDP43. Given that MDVs represent a key organellar surveillance pathway and intercellular signaling conduit, we isolated extracellular vesicle (EV) fractions from normal and degenerated NP cells. Immunoblotting revealed enriched TDP43 within EVs derived from degenerated NP cells relative to controls (Fig. 6a). Although nanoparticle tracking analysis confirmed similar vesicle size distributions between groups (Supplementary Fig. 4a), nanoscale flow cytometry (NanoFCM) demonstrated significantly elevated proportions of TDP43^+^ EVs in the degenerated group (Fig. 6b). Besides, the IL-1β stimulation increased both TDP43 content within EVs (Fig. 6c) and total EV yield, despite comparable size profiles (Supplementary Fig. 4b). NanoFCM demonstrated significantly elevated proportions of TDP43^+^ EVs in the IL-1β-treated group (Fig. 6d). To determine the subcellular origin of vesicular TDP43, we depleted TOMM22^+^ MDVs from EV fractions using immunomagnetic beads (Fig. 6e). Post-depletion efficacy was validated by diminished TOMM22⁺ EV proportions (Supplementary Fig. 4c). Crucially, TDP43 content in degenerated NP-derived EVs concurrently decreased (Fig. 6f), indicating preferential packaging of mitochondrial TDP43 into MDVs.

MIRO1/MIRO2, the Rho GTPases, were implicated in MDV biogenesis and cargo transportation^20^. Our Co-IP results confirmed physical interactions between MIRO1/MIRO2 and TOMM22 in both normal and degenerated NP cells (Fig. 6g). This interaction was spatially corroborated through subcellular colocalization via immunofluorescence (Fig. 6h). Upon functional impairment of MIRO1/MIRO2, subsequent isolation of EVs revealed significantly diminished incorporation of TOM22 and TDP43 into the vesicular fraction compared with the control group (Fig. 6i). Consistent with this result, NanoFCM quantified a marked reduction in TDP43^+^ EV subpopulations in the MIRO1/MIRO2 knockdown groups (Fig. 6j). These findings conclusively establish MDVs as a regulated disposal route for mitochondrial TDP43, orchestrated through the MIRO1/MIRO2-directed cargo encapsulation.

### Effects of TDP43-loading MDVs on cellular inflammation and senescence during NP cell degeneration

**Fig. 7:**
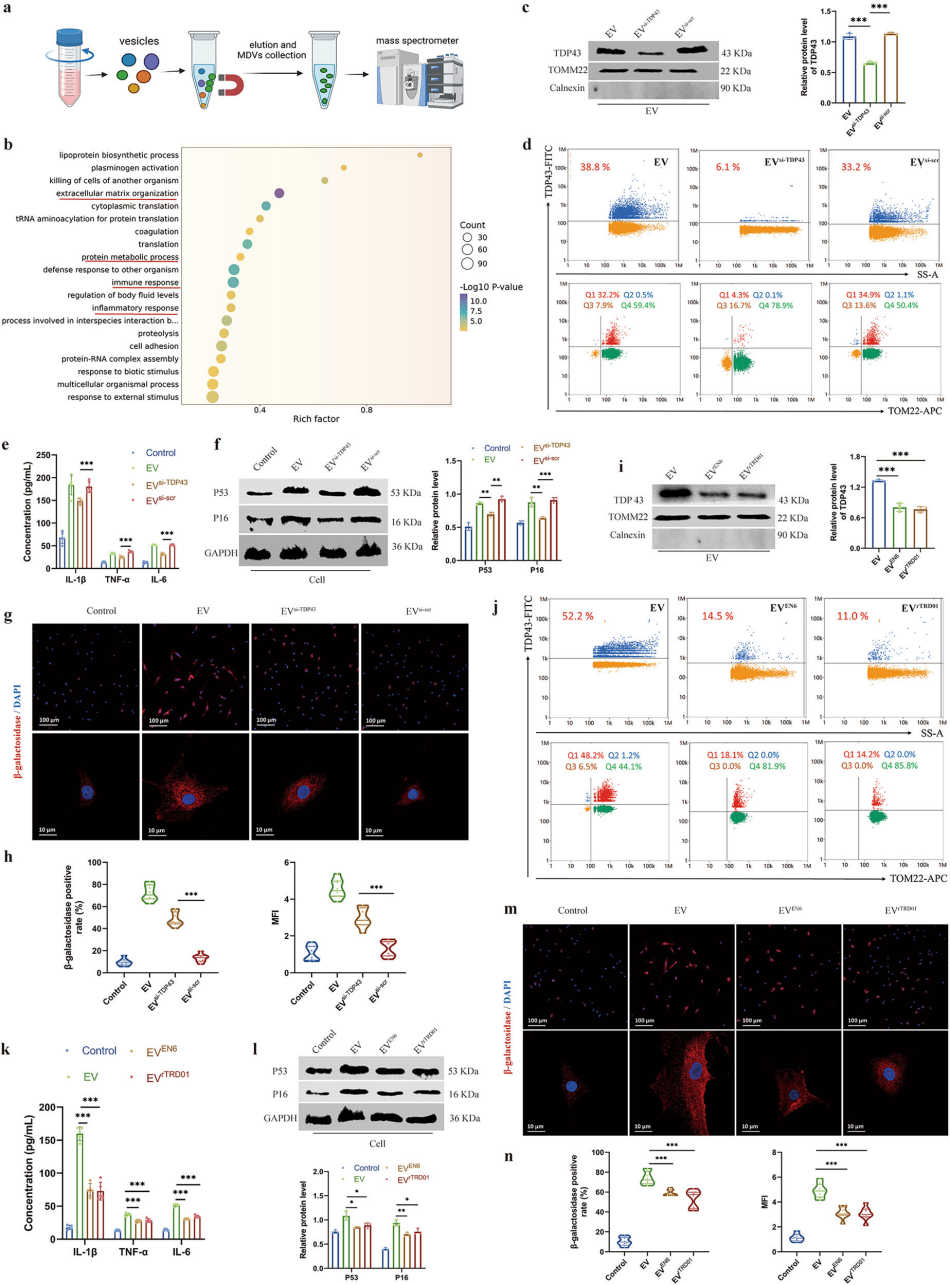
TDP43-loading MDVs promote cellular inflammation and senescence. **a** Workflow of mitochondrial-derived vesicle (MDV) isolation and analysis. **b** GO enrichment analysis of differentially expressed MDV proteins (degenerative nucleus pulposus (NP) versus normal-NP). **c** Western blot and relative protein levels of TDP43 and TOMM22 in extracellular vesicle (EV) fractions. EVs are from NP cells transfected with si-TDP43 or si-scr (EV^si-TDP43^ or EV^si-scr^). **d** Nanoscale flow cytometry analysis of TDP43-FITC and TOMM22-APC in EV fractions. **e** Concentration of secreted IL-1β, TNF-α, and IL-6 in NP cells treated with EV^si-TDP43^ or EV^si-scr^. **f** Western blot and relative protein levels of P53 and P16 in respective groups. **g**,**h** Immunofluorescence images, positive cell rate, and mean fluorescence intensity (MFI) of β-galactosidase in respective groups. **i** Western blot and relative protein levels of TDP43 and TOMM22 in EV fractions. EVs are from NP cells treated with EN6 or rTRD01 (EV^EN6^ or EV^rTRD01^). **j** Nanoscale flow cytometry analysis of TDP43-FITC and TOMM22-APC in EV fractions. **k** Concentration of secreted IL-1β, TNF-α, and IL-6 in NP cells treated with EV^EN6^ or EV^rTRD01^. **l** Western blot and relative protein levels of P53 and P16 in respective groups. **m**,**n** Immunofluorescence images, positive cell rate, and MFI of β-galactosidase in respective groups. Data are shown as mean ± SD (*n* = 3 independent experiments). *P < 0.05, **P < 0.01, ***P < 0.001 by one-way analysis of variance. DAPI, 4′,6-diamidino-2-phenylindole; FITC, fluorescein isothiocyanate; tRNA, transfer RNA.

Using TOMM22 immunomagnetic isolation coupled with quantitative mass spectrometry (Fig. 7a), we discerned 393 upregulated and 387 downregulated proteins in MDVs from degenerated NP cells versus healthy controls (Supplementary Fig. 5a). Bioinformatic enrichment analysis revealed that these differentially expressed proteins were predominantly secretory proteins associated with extracellular space, ECM, and multivesicular bodies (Supplementary Fig. 5b), with a prominent representation of mitochondrial components closely linked to mitochondrial functions (Supplementary Fig. 5c). GO analysis further implicated these MDVs in ECM disassembly, protein catabolism, and inflammatory cascades (Fig. 7b) — core pathways driving IDD, suggesting their active involvement in disease progression. Notably, degenerative cell-derived MDVs triggered dose-dependent secretion of pro-inflammatory cytokines (IL-1β, TNF-α, and IL-6) and upregulated senescence markers P53/P16 in recipient NP cells (Supplementary Fig. 6a,b). These effects were corroborated by elevated β-galactosidase activity and G1/G0-phase arrest (Supplementary Fig. 6c,d). This suggests that MDVs derived from degenerated NP cells promote cellular senescence and exacerbate inflammatory responses.

To dissect TDP43-specific pathology, we generated MDVs from TDP53-depleted NP cells (EV^si-TDP43^) via siRNA transfection. EV^si-TDP43^ exhibited substantially diminished TDP43 cargo (Fig. 7c) and reduced TOMM22^+^/TDP43^+^ EV subpopulations (Fig. 7d). Critically, EV^si-TDP43^ attenuated pro-inflammatory cytokine secretion (Fig. 7e), suppressed P53/P16 expression (Fig. 7f), reduced β-galactosidase positivity (Fig. 7g,h), and mitigated G1/G0 arrest (Supplementary Fig. 6e) compared with control EVs. By contrast, we treated NP cells with two inhibitors of TDP43 and obtained their MDV components (EV^EN6^ and EV^rTRD01^, respectively). Compared with control EVs, both EV^EN6^ and EV^rTRD01^ exhibited significantly reduced TDP43 cargo levels (Fig. 7i) and decreased proportions of TDP43⁺ EVs (Fig. 7j). Moreover, these MDVs attenuated pro-inflammatory cytokine secretion in recipient NP cells (Fig. 7k), concurrently downregulated protein expression of P53 and P16 (Fig. 7l), and diminished β-galactosidase-positive cell ratios with attenuated fluorescence intensity (Fig. 7m,n). Flow cytometry further demonstrated alleviated G1/G0 phase arrest and restored S-phase progression in the EV^EN6^ and EV^rTRD01^ groups (Supplementary Fig. 6f). Collectively, these findings reveal that degenerated NP cells release TDP43-enriched MDVs, which act as intercellular pathogenic mediators that propagate inflammatory senescence and exacerbate disc degeneration.

## Discussion

**Fig. 8:**
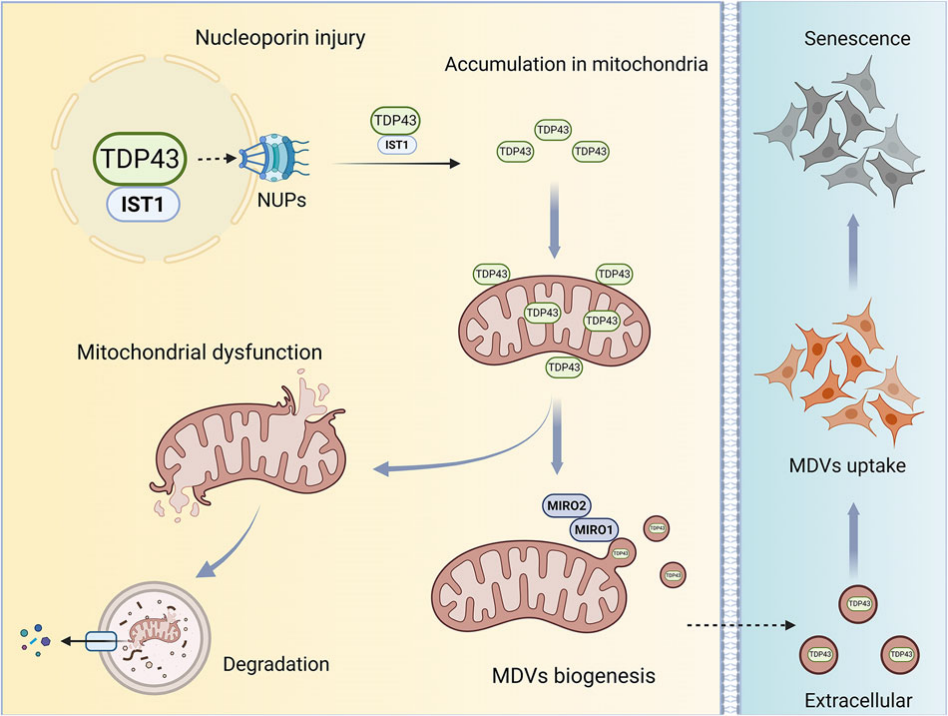
TDP43 cytoplasmic localization promotes mitochondrial dysfunction and intervertebral disc degeneration. The schematic representation illustrates the mitochondrial accumulation of TDP43 and elucidates the biogenesis mechanism of TDP43-containing mitochondrial-derived vesicles (MDVs) subsequent to their internalization by NP cells to elicit disc degeneration.

This study identified TDP43 as a critical mediator in IDD through comparative transcriptomic analysis of degenerated and stressed versus normal NP cells. Elevated TDP43 expression in degenerated human and animal NP tissues was found to correlate with the severity of disc degeneration. Mechanistically, aberrant cytosolic location, specifically mitochondrial accumulation of TDP43, emerged as a pivotal contributor to NP cell dysfunction. The mitochondrial mislocalization of TDP43 was associated with nuclear pore complex impairment, leading to MMP collapse and cellular senescence. Notably, TDP43-containing MDVs were identified as a potential mitochondrial quality control pathway, facilitating extracellular export of cytosolic TDP43. However, these TDP43-loaded MDVs propagated inflammatory phenotypes and accelerated senescence in recipient NP cells. Importantly, inhibition of TDP43 alleviated mitochondrial dysfunction and senescence in NP cells, highlighting its therapeutic potential as a molecular target for IDD intervention (Fig. 8).

IDD represents a multifactorial degenerative process involving intricate biomechanical and molecular interactions^21^. As the primary shock-absorbing structure of the spine, disc degeneration is closely associated with chronic low back pain and neurological compression symptom^22^. Cumulative evidence implicates diverse stress stimuli in IDD pathogenesis. Chronic mechanical overloading disrupts ECM homeostasis by destroying the balance between synthesis and degradation of collagen fibers and proteoglycans^23,24^. Oxidative stress, characterized by excessive reactive oxygen species accumulation, inflicts macromolecular damage to DNA, proteins, and lipids within NP cells, while concurrently activating pro-inflammatory cascades (for example, IL-1β and TNF-α) that exacerbate ECM catabolism^25–27^. Additional stressors including nutrient deprivation, acidic microenvironments, and endoplasmic reticulum stress collectively drive NP cell dysfunction toward apoptotic and senescent phenotypes, ultimately accelerating structural disintegration^28–30^. The synergistic effects of these stressors create a self-perpetuating cycle of cellular homeostasis disruption, propelling irreversible IDD progression. The present study focuses on mitochondrial damage related to TDP43. It reveals that the accumulation of TDP43 in mitochondria can lead to mitochondrial dysfunction. Mitochondrial dysfunction has been demonstrated to induce cellular senescence and the secretion of pro-inflammatory cytokines, which are consistent with the changes in degenerated NP cells under various stress environments, as outlined in the extant literature^31,32^. In summary, the molecular mechanisms underlying IDD are complex and require further in-depth exploration to uncover additional potential mechanisms, thereby facilitating the identification of effective therapeutic targets.

RBPs, master regulators of post-transcriptional gene expression, have recently emerged as critical players in IDD pathophysiology^13^. Through modulation of RNA stability, localization, and translational efficiency, RBPs orchestrate cellular stress responses, proliferation, and apoptosis^33^. Dysregulated RBP expression in degenerated discs correlates with aberrant stress signaling. According to Shao et al.^34^, human antigen R (HuR) prompted NKRF mRNA stability via binding to its AU-rich elements, thereby suppressing inflammation and ECM degradation in NP cells. NP cells with HuR deficiency are predisposed to undergoing an inflammatory phenotype^34^. The RBP FUS was also found to bind to recombinant growth factor GRB10 pre-mRNA to regulate the circRNA synthesis, which regulates the ECM metabolism in NP cells via the circRNA–miRNA axis^35^. However, chronic stress induces pathological RBP aggregation into stress granules, disrupting nucleic acid metabolism. FUS has been observed to form liquid compartments at sites of DNA damage and in the cytoplasm in response to stress^36^. These liquid-like compartments underwent aberrant phase transitions and formed aggregates, which was central to the development of amyotrophic lateral sclerosis^36^. Cytoplasmic aggregation of TDP43 is a prevalent pathological hallmark of amyotrophic lateral sclerosis^37,38^. According to Wang et al., TDP43 colocalizes with and accumulates in mitochondria within human motor neurons^19^. This mitochondrial accumulation of TDP43 inhibits the translation of mitochondrial mRNAs, consequently impairing mitochondrial function and morphology. Consistent with previous findings, our present study has also elucidated the pathological role of TDP43 in NP cells. Specifically, TDP43 is implicated in cytoplasmic abnormal aggregation and disruption of nuclear pore complexes within NP cells. Furthermore, the mitochondrial aggregation of TDP43 induces a loss of MMP and mitochondrial fragmentation, ultimately leading to mitochondrial dysfunction.

TDP43, an RBP closely associated with neurodegenerative diseases, has an unclear relationship with IDD. The translocation of TDP43 from the nucleus to the cytoplasm and its subsequent aggregation causes cytotoxicity^39^. Additionally, the abnormal accumulation of TDP43 in organelles interferes with their normal functions, leading to cellular damage^40,41^. There is a strong connection between TDP43 and mitochondrial dysfunction. Research has shown that abnormal aggregation of TDP43 can cause significant alterations in mitochondrial morphology and function^19,41,42^. A study demonstrated that TDP43 could translocate into mitochondria and induce mitochondrial DNA release through the permeability transition pore, resulting in STING activation and subsequent cellular inflammation^41^. Another study further revealed that oxidative stress promotes the aggregation of TDP43 and mitochondrial proteins, which modulates gene expression via binding to various miRNAs and ultimately exacerbates mitochondrial stress^42^. Besides, TDP43 exhibits a preferential binding affinity for mitochondria-transcribed RNAs, inhibiting their translation^19^. Concurrently, mitochondrial TDP43 disrupts the assembly of oxidative phosphorylation complex I and impairs mitochondrial morphology^19^. Mitochondria, often referred to as the powerhouse of the cell, have a critical role in energy production^43^. Dysfunction in mitochondria severely impacts cellular energy supply, which is closely associated with cellular senescence^44,45^. Our study revealed that TDP43 preferentially binds to mitochondrial RNAs, including ND6, COXI, and COXIII, in degenerated NP cells. Moreover, the accumulation of mitochondrial TDP43 leads to the loss of MMP and induces cell cycle arrest in NP cells. Inhibiting TDP43 through genetic or pharmacological approaches, or preventing its cytoplasmic localization, effectively mitigates NP cell senescence. These findings highlight the significance of restoring mitochondrial function in replenishing cellular energy supply and decelerating cellular senescence. Targeting the modulation of mitochondrial function may emerge as a promising therapeutic strategy for IDD.

MDVs, membrane-bound vesicles budding from mitochondria, serve as the mechanism of mitochondrial quality control, which selectively encapsulate damaged components for lysosomal degradation or extracellular export via tightly regulated pathways^46^. Unlike mitophagy, which involves the degradation of entire mitochondria, MDVs facilitate the targeted disposal of specific mitochondrial cargo, such as oxidized proteins or lipids, thereby preserving mitochondrial integrity under stress conditions^47,48^. The formation of MDVs is negatively regulated by PINK1 and Parkin, which are also implicated in mitophagy^49–51^. A study has suggested that Parkin blocks the formation of MDVs by directing damaged mitochondrial content to lysosomes^51^. Importantly, the formation of MDVs appears to precede mitophagy, suggesting that it serves as an early protective response to mitochondrial damage^52^. Dysregulation of MDV biogenesis has been linked to various pathological conditions, including neurodegenerative diseases and cardiac dysfunction, highlighting its significance in maintaining cellular homeostasis^49,53^. The precise mechanisms underlying MDV biogenesis remain poorly understood. However, recent studies have shed light on this process, demonstrating that MIRO1/2 facilitates MDV formation through the generation of membrane protrusions along microtubule filaments. The depletion of either MIRO1 or MIRO2 resulted in a significant decrease in the number of MDVs. This process is subsequently followed by the recruitment of GTPase DRP1, which has a crucial role in mediating mitochondrial membrane scission^20^. Todkar et al.^51^ also found that optic atrophy 1 and sorting nexin 9 are involved in the cargo sorting into MDVs, especially targeting damaged mitochondrial content to MDVs. Our results show a marked increase in TDP43 levels in MDVs from degenerated NP cells. This upregulation aligns with the elevated TDP43 expression observed within degenerated NP cells, indicating a potential role in mitochondrial quality control regulation. TDP43-aggragated mitochondria may selectively release MDVs to decrease the TDP43 content. Further investigation is required to clarify the molecular mechanisms underlying MDV formation and their interaction with other mitochondrial quality control pathways. Understanding these processes may provide novel therapeutic targets for diseases associated with mitochondrial dysfunction.

MDVs have an important role in intercellular communication and cellular homeostasis, influencing both the originating cells and the surrounding microenvironment. Within the host cell, MDVs serve as a selective mechanism for removing damaged mitochondrial components, thereby preventing the accumulation of toxic materials and maintaining mitochondrial function^46^. Beyond the host cell, MDVs can be released into the extracellular space, where they exert paracrine effects on neighboring cells^54^. These vesicles carry mitochondrial components and other molecules, which can modulate cellular biological processes such as inflammation, metabolism, and stress responses^54–56^. For instance, MDVs containing mitochondrial DNA have been shown to activate innate immune pathways in recipient cells, which depend on sorting nexin 9 (ref. ^55^). Therefore, it is evident that MDVs serve as crucial intercellular signaling mediators, facilitating the transmission of mitochondrial signals to neighboring cells as well as to distant tissues and organs. A study further demonstrated that MDVs containing mitochondrial damage-associated molecular pattern, including *N*-formyl peptides, mitochondrial DNA, and oxidized proteins, have a significant role in triggering specific pro-inflammatory cytokine responses in recipient cells^51^. Our study indicates that MDVs containing TDP43, likely derived from mitochondrial TDP43 aggregates, drive recipient NP cells toward a pro-inflammatory phenotype. We found that TDP43-laden MDVs induce cell cycle arrest in NP cells and stimulate the secretion of key inflammatory cytokines such as TNF-α, IL-1β, and IL-6. These findings suggest that MDVs released from degenerated NP cells disrupt the normal function of adjacent NP cells, thereby contributing to the progression of IDD. Therefore, in-depth research into the mechanisms of MDVs and mitochondrial dysfunction is of significant importance for understanding the pathophysiological processes of IDD. Further studies are needed to clarify the molecular composition of MDVs, their mechanisms of release, and their functional impact on recipient cells, which may reveal new therapeutic strategies for diseases linked to mitochondrial dysfunction.

This study has several unresolved questions meriting further investigation. First, the precise mechanism by which TDP43 orchestrates mitochondrial mislocalization and nuclear pore impairment remains incompletely defined — particularly regarding potential cofactors regulating its pathological translocation. Second, although TDP43-loaded MDVs propagate inflammatory senescence in vitro, their intercellular trafficking efficiency and biodistribution in vivo were not quantitatively mapped. It is essential to develop MDV secretion inhibitors for in vivo applications to enhance the reliability of experimental outcomes in animal models. Third, the downstream molecular events of vesicular TDP43 transfer require further deconvolution, particularly regarding its interaction with established IDD mediators. Addressing these gaps would strengthen therapeutic translation. Future investigations will rigorously delineate the functional role and pathogenic mechanisms of TDP43 in IDD.

In conclusion, this study elucidates TDP43 as a master regulator orchestrating mitochondrial dysfunction, cellular senescence, and inflammatory cascades in IDD. Mitochondrial mislocalization of upregulated TDP43 impairs nuclear pore integrity and triggers mitochondrial dysfunction in NP cells, culminating in irreversible senescence. Crucially, TDP43 hijacks mitochondrial quality-control pathways to enter MDVs, converting these vesicles into vehicles for cross-cellular senescence and inflammation transmission. Therapeutically, targeted inhibition of TDP43 rescued mitochondrial function and suppressed cellular senescence markers and retarded disc degeneration in vivo. These findings not only establish TDP43-loaded MDVs as pathogenic conductors of disc degeneration but also validate TDP43 intervention as a promising strategy for IDD.

## Supplementary information

Supplementary Information

## Data Availability

All data generated or analyzed during the current study are included in this published article and supplemental information.
