# Supplementary material for: TDP43 cytoplasmic mislocalization initiates mitochondrial dysfunction and intercellular senescence propagation in intervertebral disc degeneration
Source: Exp Mol Med. 2026 May 1;58(5):1464–78. doi: 10.1038/s12276-026-01709-z (PMC13233872; doi:10.1038/s12276-026-01709-z)
Supplement: Supplementary file 1 — Supplementary Information [file 12276_2026_1709_MOESM1_ESM.pdf]

Supplementary materials

TDP43 Cytoplasmic Mislocalization Initiates Mitochondrial dysfunction and Intercellular Senescence Propagation in Intervertebral Disc Degeneration

Supplementary methods

Animal experiments

All experimental procedures involving animals were formally approved by the Institutional Animal Care and Use Committee at Tongji Medical College, Huazhong University of Science and Technology. Animal husbandry and experimental protocols were conducted in strict accordance with the ARRIVE guidelines and institutional biosafety standards. All animals were grouped according to a random number table method. A surgical disc degeneration model of Sprague-Dawley (SD, male, 8-week-old, 250 g) rats was conducted by needle puncture. The intervertebral discs of rats (Co 8/9) were marked by palpation and examined by radiography. The sham disc was punctured with a 33-gauge needle. The degeneration (IDD) group was punctured with a 22-gauge needle. For drug injection, the Co 8/9 disc was punctured by a 22-gauge needle, after which EN6 (50 μM, 2 μL) or rTRD01 (100 μM, 2 μL) were injected via a 33-gauge needle. For plasmid injection, the TDP43 plasmid or control plasmid (5μg, 2 μL) was injected via a 33-gauge needle. The injection of drugs or plasmids was conducted on a weekly basis for a period of eight weeks.

Imaging assessment

Radiographic evaluation of intervertebral discs was conducted using an in vivo MS FX PRO imaging system (Bruker, USA). The μCT was conducted using μCT scanning system (SkyScan 1176, Bruker) and then three-dimensionally restricted by CT-Vox software (Bruker). Disc height measurements were obtained to calculate the disc height index (DHI), with degenerative changes quantified using the formula: DHI (%) = (postoperative DHI / preoperative DHI) × 100%, where postoperative and preoperative DHI values were measured following and prior to surgical intervention, respectively. Magnetic resonance imaging (MRI) was performed on a BRUKER BioSpec system (Germany), with sagittal T2-weighted sequences employed to assess disc hydration status through signal intensity analysis. Disc degeneration was further classified using Pfirrmann grading (Grade I: normal; Grade V: severe degeneration) based on T2-weighted MRI findings.

Western blotting

Cellular proteins and extracellular vesicles (EVs) proteins were lysed using RIPA buffer (Beyotime Biotechnology, Shanghai, China). Subcellular fractionation was performed with a Nuclear and Cytoplasmic Protein Extraction Kit (Beyotime Biotechnology) according to the manufacturer's protocol. The protein sample (30 μg per lane) was subjected to sodium dodecyl sulfate polyacrylamide gel electrophoresis (0.45 μm, SDS-PAGE) and subsequently transferred onto PVDF membranes (Millipore, MA, USA). The bands were then washed in blocking buffer (Epizyme, Shanghai, China) for 30 minutes and then incubated with a diluted primary antibody overnight. Following this, the bands were washed with TBST buffer and then incubated with HRP-conjugated secondary antibody (Proteintech, Wuhan, China) for one hour. Enhanced chemiluminescence detection was conducted using ECL Prime (Thermo Fisher

Scientific, USA) on a ChemiDoc MP imaging system (Bio-Rad, CA, USA). Immunoreactive bands were quantified by densitometry using ImageJ 1.52a software (NIH, USA) with GAPDH as loading control. Triplicate independent experiments were performed for statistical validation.

Quantitative real-time polymerase chain reaction

Gene expression profiling was performed using SYBR Green-based quantitative real-time polymerase chain reaction (RT-qPCR). Cellular RNA was extracted with TRIzol™ Reagent (Invitrogen, CA, USA) through standard phenol-chloroform phase separation. RNA sample was converted to cDNA using RT SuperMix (Vazyme Biotech, Nanjing, China) and PCR reactions were conducted using SYBR qPCR Master Mix (Vazyme Biotech) on a Real-Time quantitative PCR System (Bio-Rad, CA, USA). Relative mRNA expression levels were calculated with GAPDH as endogenous control. Experimental data derived from three biological replicates were subjected to statistical analysis.

RNA sequencing and data analysis

Cellular RNA was extracted with TRIzol™ Reagent (Invitrogen, CA, USA) through standard phenol-chloroform phase separation. Following rigorous quality control assessment, qualified RNA samples were subjected to stranded mRNA-seq library preparation. Sequencing was performed on NovaSeq 6000 platform (Illumina) with 150 bp paired-end reads, and then mapped to the human genome using STRA 2.5 software. Differential gene expression analysis was conducted through edgeR (v3.32.1) with thresholds (P-value cutoff of 0.05; fold-change cutoff of 2). Functional enrichment analysis of differentially expressed genes (DEGs) was performed using KOBAS (v2.1.1) with dual filtering criteria ($P<0.05$, $FDR<0.05$) against Gene Ontology (GO) biological process database and KEGG pathway repository.

Immunofluorescence

Samples were initially fixed with 4% paraformaldehyde (15 min) followed by permeabilization using 0.2% Triton X-100 (10 min). Non-specific binding sites were blocked with 2% goat serum albumin (1 h), after which samples underwent three 5-min washes with PBS-T buffer. Primary antibody incubation was conducted at 4°C for 12 h under humidified conditions. Subsequent detection employed species-matched CoraLite-conjugated secondary antibodies (Proteintech, Wuhan, China) with 1 h incubation in light-protected environment. Nuclear counterstaining was achieved using DAPI (5 μg/ml, Beyotime Shanghai, China) for 5 min. The fluorescent images were systematically acquired from randomly selected fields using a microscope (Olympus, PA, USA) operated by three independent blinded investigators. Quantitative analysis of fluorescence intensity was performed through ImageJ 1.52a software (NIH, Bethesda, MD, USA) with background subtraction and threshold standardization across all samples.

Histological evaluation

Following euthanasia, harvested intervertebral discs underwent fixation in 4% formaldehyde for 48 hours and decalcification in 10% EDTA for 30 days. Processed specimens were subsequently dehydrated, paraffin-embedded, and sectioned into 4-μm slices. Tissue sections were stained with hematoxylin and eosin (HE), Safranin O-fast green (S-O), or Masson for histological evaluation. Degeneration severity was histologically scored using a 15-point scale (0: normal morphology; 15: severe degeneration), incorporating assessments of five distinct pathological features: Grade I, 0-3 points; Grade II, 4-7 points; Grade III, 8-11 points; Grade IV, 12-15 points. Immunofluorescence was performed on deparaffinized, rehydrated sections after microwave-mediated antigen retrieval in sodium citrate (15 min). Sections were blocked with 5% BSA (30 min), incubated with TDP43 primary antibody overnight, and subsequently treated with fluorescein-conjugated secondary antibody. Images at random views were obtained under a microscope (Olympus, USA) by three independent researchers. The mean fluorescent intensity were quantified using ImageJ 1.52a (National Institutes of Health, USA).

Mitochondria and MDVs isolation

Mitochondria were isolated using the Cell Mitochondria Isolation Kit (Beyotime, Shanghai, China) following standard protocols. Briefly, adherent cells were digested with 0.25% trypsin-EDTA and pelleted by centrifugation at 200 × g for 5 min at room temperature. Approximately 2 × $10^{\wedge 7}$ cells were resuspended in 1 ml ice-cold mitochondrial isolation reagent containing protease inhibitors. After 10 min of incubation on ice, cell suspension was homogenized and the homogenate was collected. The homogenate was subjected to centrifugation at 11,000 × g for 10 min (4°C). The final pellet containing purified mitochondria was resuspended in mitochondrial storage buffer. The MDV-containing supernatant was used to purify MDVs. The supernatant was centrifugation at 15 000 × g for 10 min and then at 110000 × g for 70 min to obtain the MDVs pellet. Protein concentration was quantified using a bicinchoninic acid (BCA) assay kit (Thermo Fisher Scientific, USA). The mitochondrial proteins or MDVs were then conducted to the Western blotting analysis. Morphological analysis of MDVs was performed by transmission electron microscopy (TEM, FEI Tecnai G20 TWIN, USA). Quantitative assessment utilized nanoparticle tracking analysis (NTA, NANOSIGHT NS300, Malvern, UK) and the final particle concentration (particles/mL) was calculated using the NTA software.

RNA and plasmid transfection

Small interfering RNA (siRNA) and scrambled siRNA (si-scr) were chemically synthesized and dissolved in diethyl pyrocarbonate (DEPC)-treated water at a working concentration of 20 μM. The plasmids (WT-TDP43, mutant TDP43 and control NC plasmid) were custom-synthesized and sequence-verified by General Biology (Anhui, China). Cells seeded in 24-well plates were grown to 50% confluence prior to transient transfection. RNA oligonucleotides (100 nM) or 5 μg plasmid DNA were mixed with Lipo3000 transfection reagent (BioSharp, Beijing, China) in serum-free Opti-MEM (Gibco, USA) following the manufacturer's protocol. Following 6 h transfection,

medium was replaced with complete growth medium. Cells were harvested at 24 h post-transfection for total RNA extraction (TRIzol) and protein lysate preparation (RIPA buffer containing protease inhibitors), followed by RT-qPCR (SYBR Green system) and Western blotting (SDS-PAGE) analysis respectively.

Protein and RNA immunoprecipitation

Co-immunoprecipitation experiments were performed using modified RIPA lysis buffer (50 mM Tris-HCl pH7.4, 150 mM NaCl, 1 mM EDTA, 1% NP-40) supplemented with 1× protease inhibitor cocktail (Beyotime, Shanghai, China). For protein interactions, lysates (500 μg) were incubated with 5 μg primary antibody or species-matched IgG control overnight at 4°C with gentle rotation, followed by 2 h incubation with protein A/G magnetic beads (MCE, Shanghai, China). The immunoprecipitates were separated using magnetic adsorption and subsequently subjected to Western blotting analysis. RNA-protein interactions were investigated through RNase-free immunoprecipitation protocol. Lysates prepared in RIP buffer (supplemented with 100 U/ml recombinant RNase inhibitor) were pre-cleared with Protein A/G beads. RNA-protein complexes were immunoprecipitated using magnetic adsorption and analyzed by TRIzol-chloroform extraction. Purified RNAs were then subjected to RT-qPCR assay.

RNA pull-down

Biotinylated mitochondrial RNAs (5 nM) were incubated with streptavidin magnetic beads (MCE, Shanghai, China) in binding buffer (20 mM Tris pH7.5, 100 mM KCl) for 1 h at 4°C. NP cell lysates (1 mg) prepared in modified buffer (50 mM Tris-HCl, 150 mM NaCl, 1% NP-40) were mixed with RNA-bound beads for 6 h at 4°C. Subsequently, the mixture was isolated using magnetic force and washed twice with washing buffer. The RNA-bound proteins were collected for Western blotting (SDS-PAGE) analysis.

Proteomic analysis

Protein samples were collected from NP cells or MDVs. For MDVs, the vesicles were incubated with Anti-TOMM22 Beads following manufacturer's protocol (Miltenyi Biotec, Germany). After elution, these MDV samples were used for subsequent detection. Proteins (30 μg per sample) were alkylated and digested with trypsin. Peptides were labeled with isobaric tags for relative and absolute quantification (iTRAQ). The iTRAQ labeling was carried out following manufacturer's protocol (Applied Biosystems Incorporation, USA). Fractionated peptides were analyzed by liquid chromatography tandem mass spectrometry (LC-MS/MS) system (Thermo Fisher Scientific, USA). MS/MS data were searched against UniProt human database using Proteome Discoverer 3.0 (FDR<1%). Differentially expressed proteins ($|\text{fold change}| \geq 1.5$, $p<0.05$) underwent DAVID GO analysis and KEGG pathway mapping (Benjamini-Hochberg correction).

Mitochondrial membrane potential analysis

Mitochondrial membrane potential (MMP) was monitored using JC-1 probe (BioSharp, Beijing, China) according to standardized protocols. Following treatment, cells were

loaded with 2 μM JC-1 in serum-free DMEM for 30 min. Cells underwent dual-wash cycles with ice-cold assay buffer. Single-cell suspensions (2×10^5 cells/ml in PBS) were analyzed on a flow cytometer (BD Biosciences, San Jose, CA, USA). Fluorescence signals were collected through 530/30 nm (green monomer) and 585/42 nm (red aggregates) filters, with compensation matrix established using unstained cells and carbonyl cyanide 3-chlorophenylhydrazone (CCCP; 10 μM)-treated controls. MMP was expressed as red/green fluorescence intensity ratio calculated using FlowJo X software (Tree Star, Inc. San Carlos, CA, USA) across three biological replicates.

Cell cycle analysis

Synchronized NP cells were harvested via trypsin-EDTA (0.25% w/v) digestion and fixed in chilled 70% ethanol at 4 °C overnight. Fixed cells were washed with PBS/1% BSA and treated with RNase A (100 μg/ml) at 37°C for 30 min, followed by propidium iodide (PI; 50 μg/ml, BioSharp) staining in darkness. Followed by filtration by 100-mesh nylon net, the samples were analyzed on a flow cytometer (BD Biosciences, San Jose, CA, USA). Cell cycle modeling was accomplished through Watson pragmatic algorithm in FlowJo X software (Tree Star, Inc. San Carlos, CA, USA), requiring G0/G1 peak CV<8% for data inclusion.

NanoFCM analysis

The MDV sample was immunolabeled with CoraLite 488 or 594-conjugated primary antibody overnight. The labeled MDV sample was then measured by a NanoFCM (U30, NanoFCM Inc, Xiamen, China). Prior to this, the instrument underwent a calibration process for particle concentration, which was conducted using 250 nm Silica Nanosphere beads. All particles that passed the detector were recorded during a 1-minute interval. Samples were then diluted in Phosphate Buffered Saline at proper ratios and administered under controlled flow with a pressure of 1.0 kPa. The subsequent analysis of the fluorescence intensity, size distribution, and particle concentration data was conducted using the NanoFCM Software (NanoFCM, Xiamen, China).

Enzyme-linked immunosorbent assay

To evaluate the pro-inflammatory cytokines released from NP cells, the supernatant was collected and centrifuged at 500 × g for 10 min. The contents of TNF-α, IL-1β or IL-6 in the supernatant were measured using corresponding Enzyme-linked immunosorbent assay (ELISA) kits (BioSharp, Beijing, China). The ELISA experiments were conducted according to the manufactural protocol. The OD value of each standard and specimen was subtracted from the OD value of the blank well. A corresponding standard curve with absorbance OD value as the y-axis (Y) and corresponding IL-1 β standard concentration as the x-axis (X) was generated. The cytokine content of the sample was converted from the standard curve to the corresponding concentration based on its OD value.

Supplementary figures

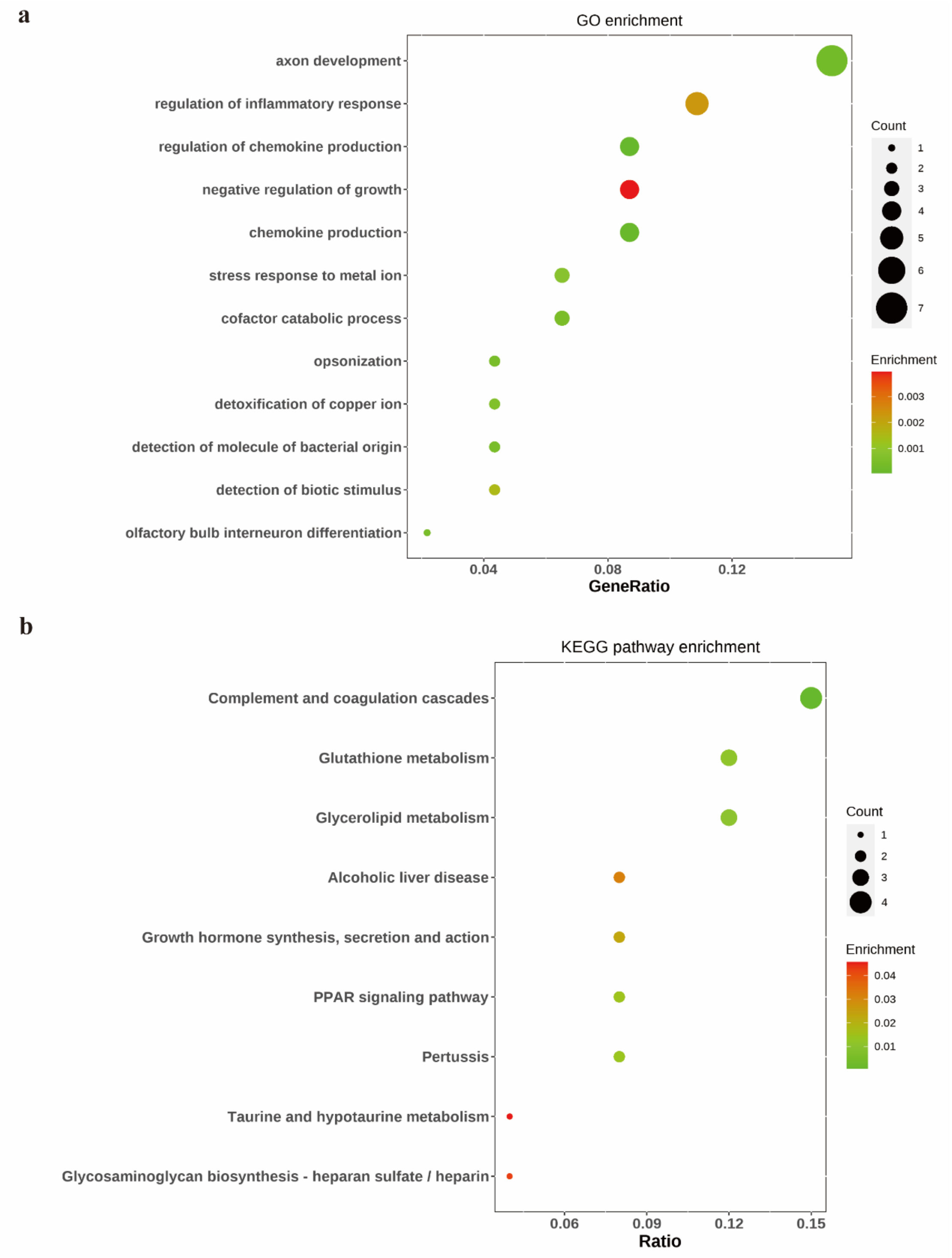

Supplementary Fig.1. GO and KEGG analysis of differentially expressed genes. a, GO enrichment analysis of differentially expressed genes in NP cells (Control vs. IL-1β and degenerative groups). b, KEGG enrichment analysis of differentially expressed genes.

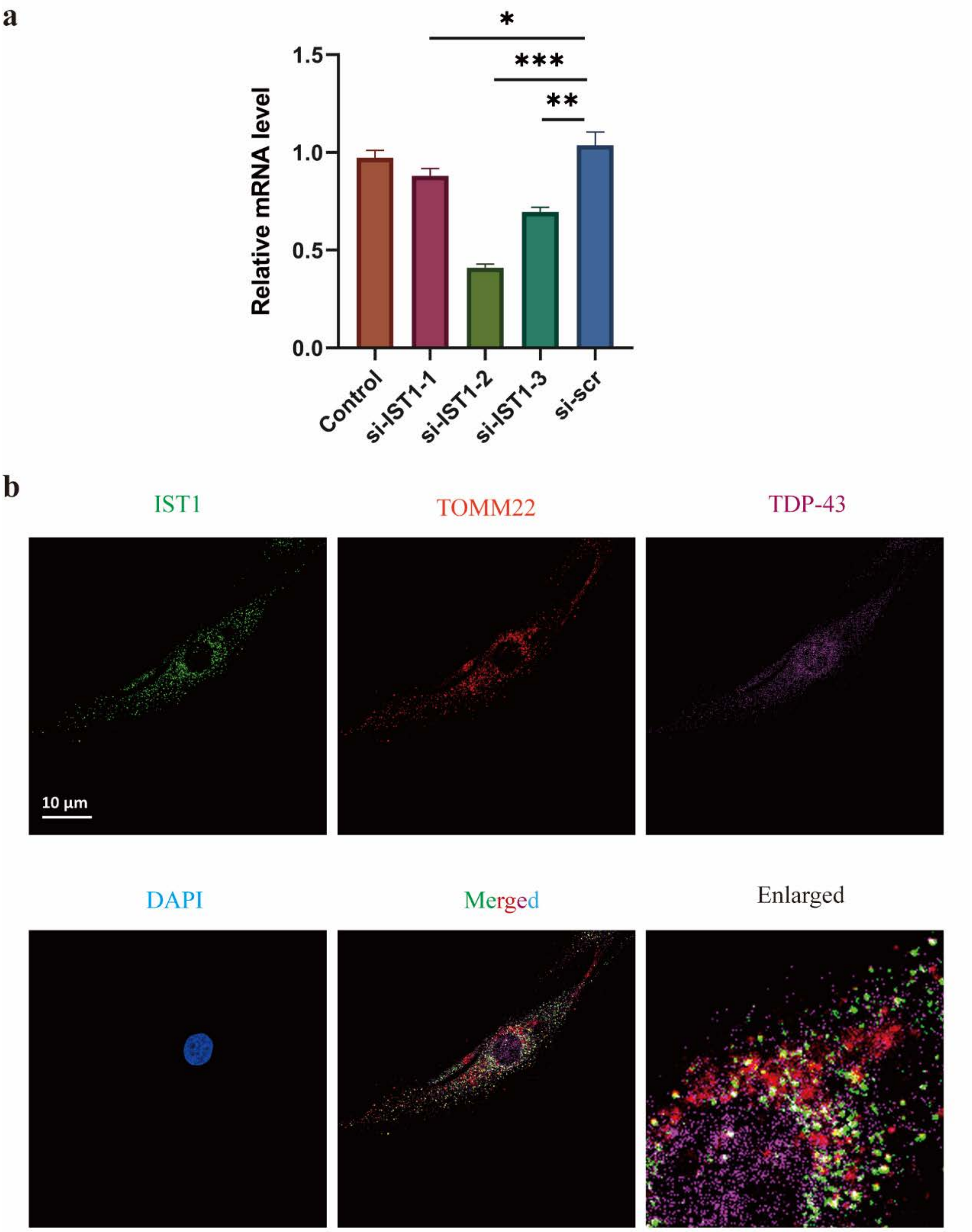

Supplementary Fig.2. The co-localization of IST1, TOMM22 and TDP43 in NP cells. a, Relative mRNA levels of IST1 in NP cells transfected with siRNAs or si-scr. b, Immunofluorescence images of IST1, TDP43, and TOMM22 in NP cells. Data are shown as the mean ± SD (n = 4). *p < 0.05, **p < 0.01, ***p < 0.001 by one way ANOVA.

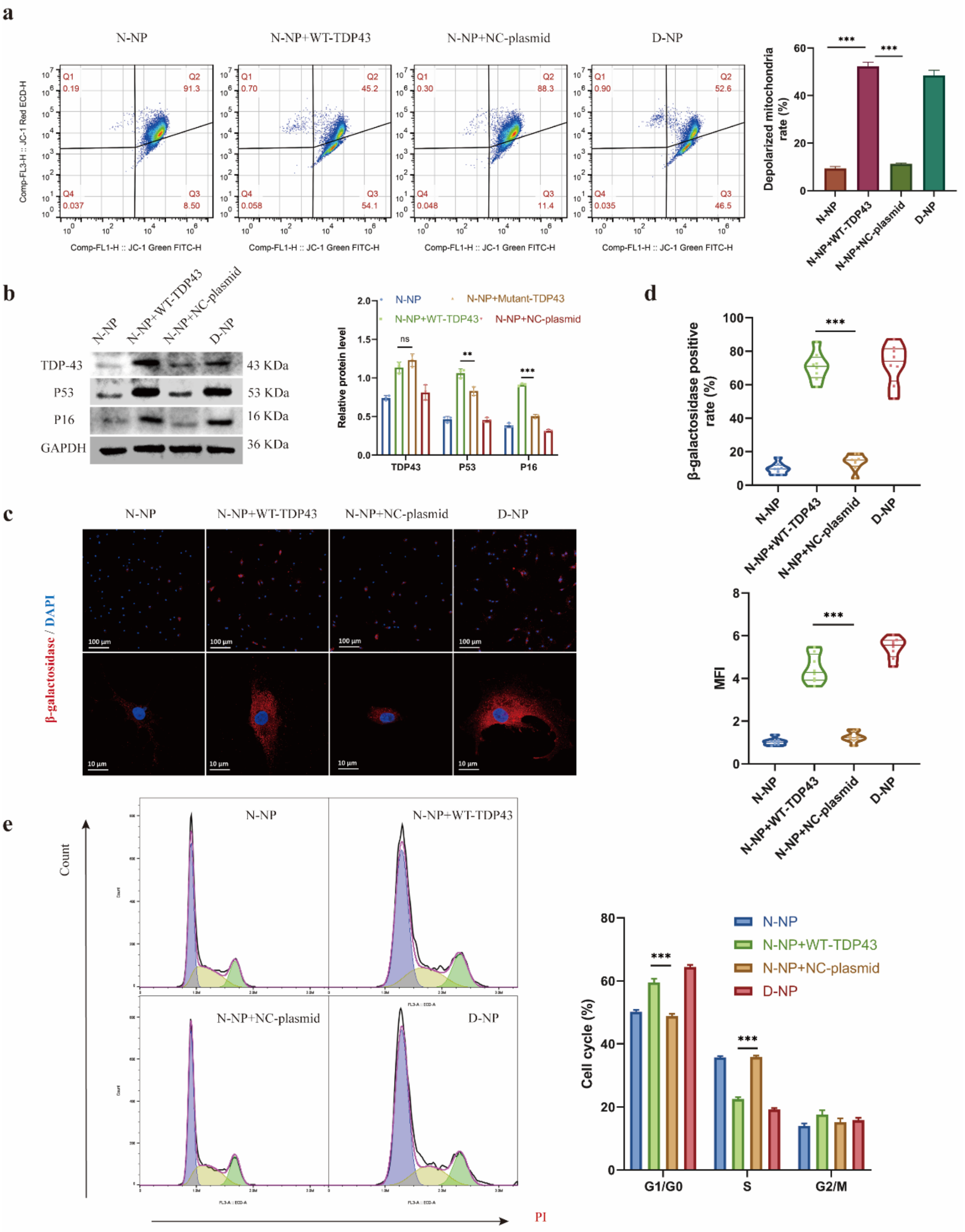

Supplementary Fig.3. TDP43 promotes mitochondrial dysfunction and NP cell senescence. a, Flow cytometry of JC-1 staining and depolarized mitochondria rate of NP cells transfected with TDP43 or NC plasmids. b, Western blot and relative protein levels of TDP43, P53, and P16 in respective groups. c-d, Immunofluorescence images, positive cell rate and mean fluorescence intensity of β-galactosidase in respective groups. e, Flow cytometry of PI staining and cell cycle modeling in respective groups. Data are shown as the mean ± SD ($n \geq 3$). **$p < 0.01$, ***$p < 0.001$, ns (not significant) by one way ANOVA.

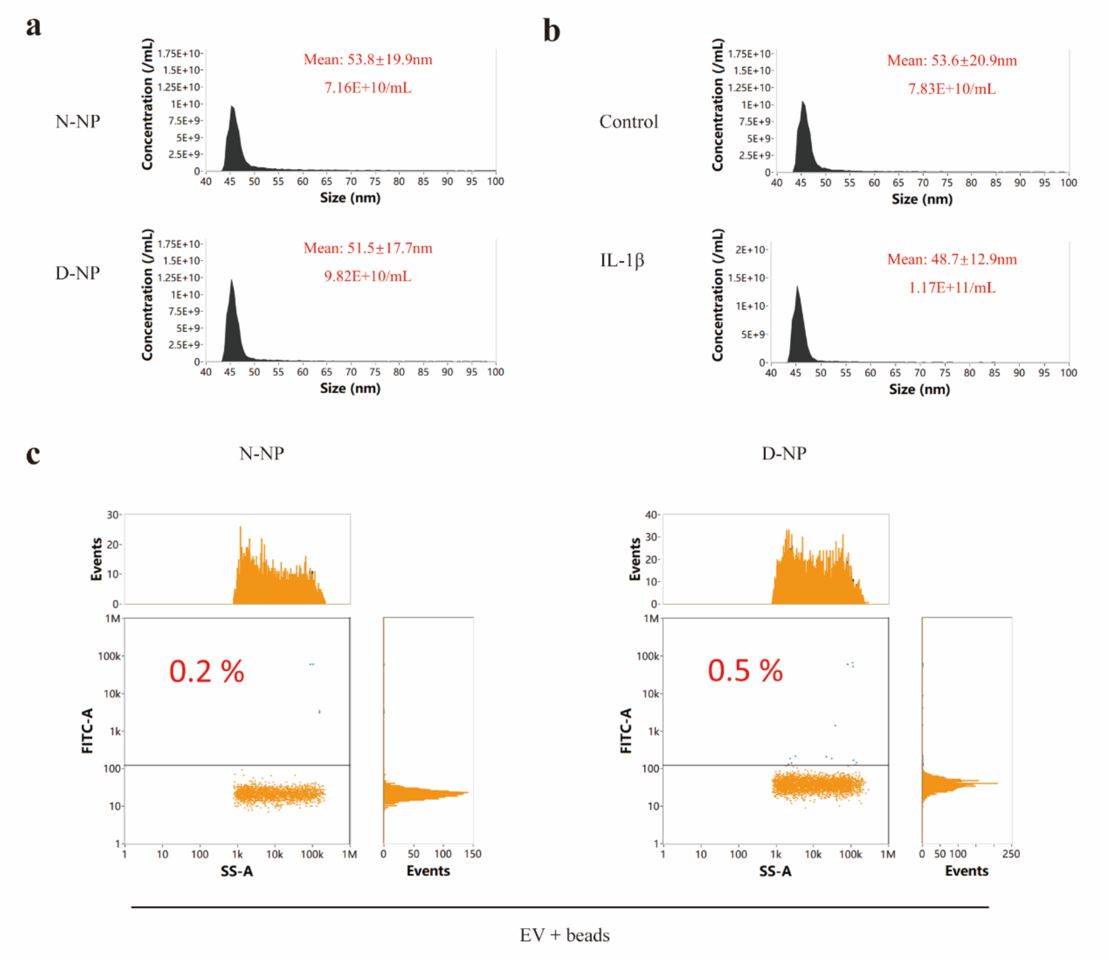

Supplementary Fig.4. Identification of EVs derived from NP cells. a, Nanoparticle tracking analysis of EVs derived from N-NP and D-NP cells. b, Nanoparticle tracking analysis of EVs derived from IL-1β-treated and control NP cells. c, NanoFCM analysis of TOMM22-FITC in EV fractions treated with TOMM22 beads.

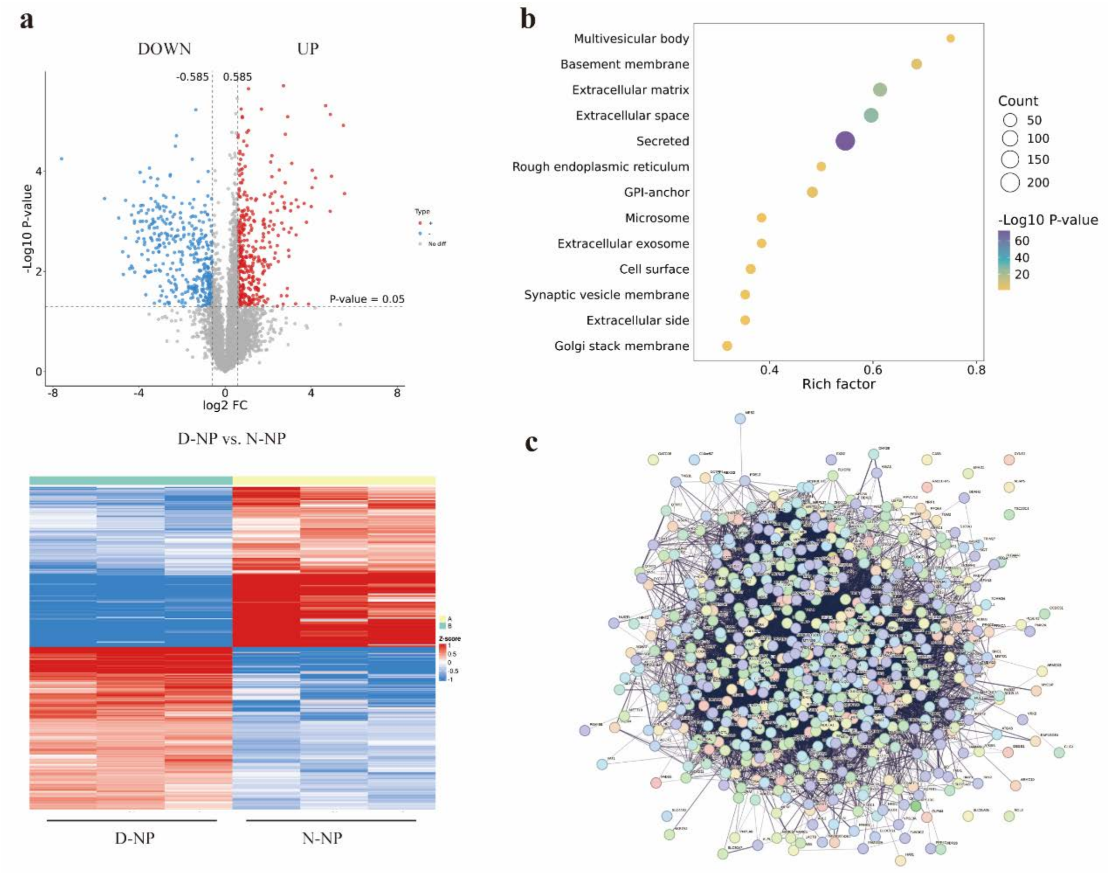

Supplementary Fig.5. Proteomics analysis of NP cell-derived MDVs. a, Volcano plot and heatmap of differentially expressed MDV proteins (D-NP vs. N-NP). b, Subcellular location analysis of differentially expressed MDV proteins. c, STRING protein interaction analysis of differentially expressed MDV proteins.

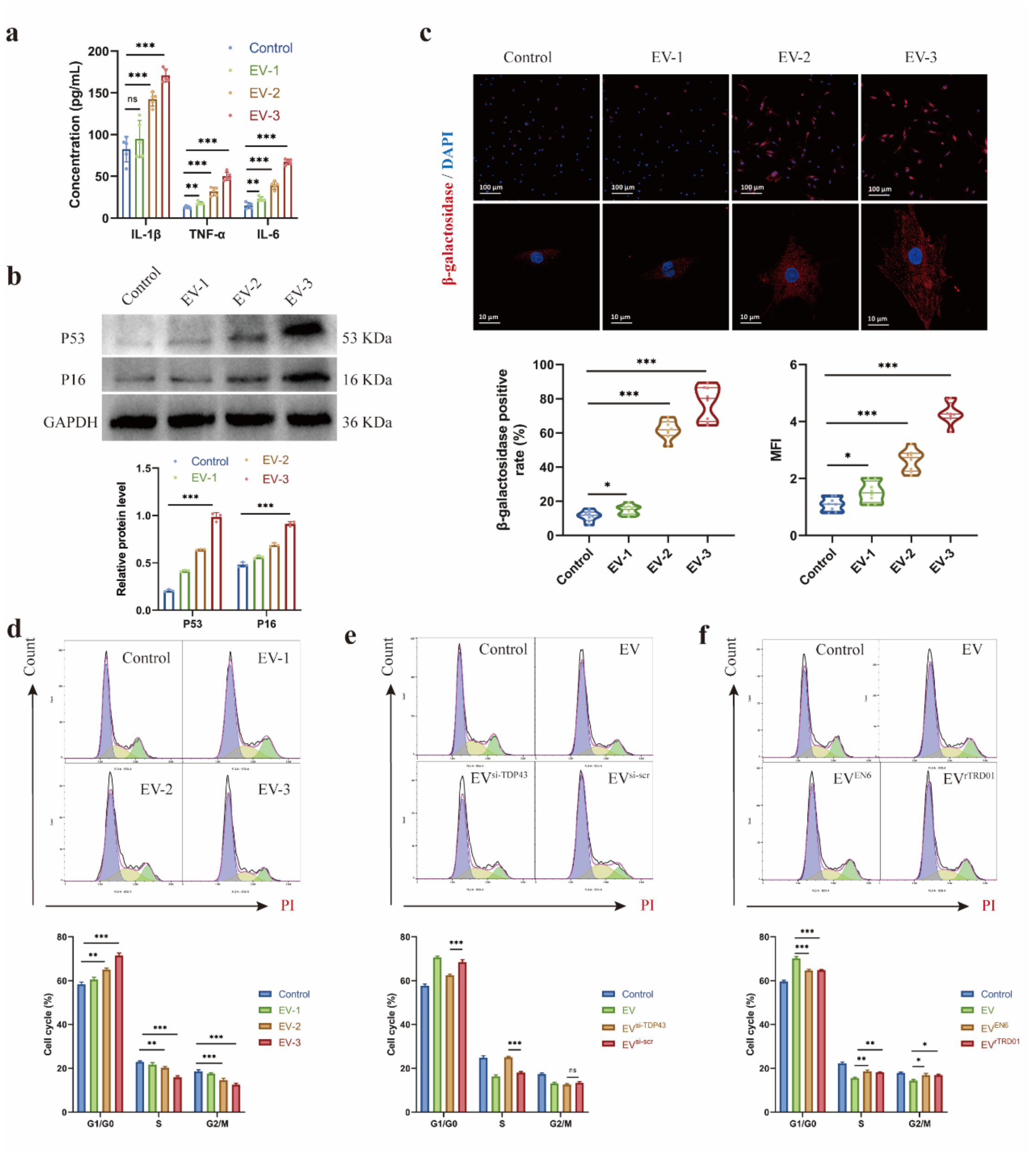

Supplementary Fig.6. MDVs induce pro-inflammatory cytokine release and cell cycle arrest in NP cells. a, Concentration of secreted IL-1β, TNF-α, and IL-6 in NP cells treated with different doses of MDVs (EV1-3). b, Western blot and relative protein levels of P53 and P16 in respective groups. c, Immunofluorescence images, positive cell rate and mean fluorescence intensity of β-galactosidase in respective groups. d, Flow cytometry of PI staining and cell cycle modeling in respective groups. e, Flow cytometry of PI staining and cell cycle modeling in NP cells treated with $EV^{si\text{-}TDP43}$ or $EV^{si\text{-}scr}$. f, Flow cytometry of PI staining and cell cycle modeling in NP cells treated with EV^{EN6} or EV^{rTRD01}. Data are shown as the mean ± SD ($n \geq 3$). *$p < 0.05$, **$p < 0.01$, ***$p < 0.001$, ns (not significant) by one way ANOVA.

Supplementary tables

Supplementary Table 1：Antibodies and reagents

Description	Source	Catalog number
Anti-TDP43	Proteintech	10782-2-AP
Anti-ACAN	Affinity Biosciences	DF7561
Anti-MMP3	Affinity Biosciences	AF0217
Anti-GAPDH	Abcam	ab8245
Anti-COXIV	Abcam	ab202554
Anti-Calnexin	Abcam	ab22595
Anti-TOMM22	Proteintech	66562-1-Ig
Anti-LAMP2	Proteintech	66301-1-Ig
Anti-NUP153	Proteintech	14189-1-AP
Anti-NUP160	Proteintech	16084-1-AP
Anti-NUP205	Proteintech	24439-1-AP
Anti-IST1	Proteintech	51002-1-AP
Anti-P53	Affinity Biosciences	AF0879
Anti-P16	Affinity Biosciences	AF5484
Anti-Alix	Proteintech	12422-1-AP
Anti-MIRO1	Proteintech	21560-1-AP
Anti-MIRO2	Proteintech	11237-1-AP
Anti-Beta Galactosidase	Proteintech	66586-1-Ig
Masson	Servicebio	G1006
Hematoxylin-Eosin	Servicebio	G1005
Safranin O-green	Servicebio	G1053
IL-1 beta	Sigma Aldrich	GF418-MG

CoraLite 488-anti-rabbit	Proteintech	RGAR002
CoraLite 594-anti-mouse	Proteintech	RGAM004
CoraLite488-conjugated TDP-43	Proteintech	CL488-80001
CoraLite594-conjugated TOMM22	Proteintech	CL594-66562
DAPI	Beyotime	P0131
Protein A/G Magnetic Beads	MedChemExpress	HY-K0202
Streptavidin Magnetic Beads	MedChemExpress	HY-K0208
rTRD01	MedChemExpress	HY-153898
EN6	MedChemExpress	HY-128892
Type II collagenase	MedChemExpress	HY-E70005B
Fetal bovine serum	Cell-Box	AUS-01S-02

Supplementary Table 2：Primers of targeted genes

Description	Forward	Reverse
ND1	TCAAACTCAAACTACGCCCTG	GTTGTGATAAGGGTGGAGAGG
ND2	ACCAAATCTCTCCCTCACTAAACG	CCACCTCAACTGCCTGCTATG
ND3	CCCTTACGAGTGCGGCTTC	AGTGGCAGGTTAGTTGTTTGTAGG
ND4	AGCCCTCGTAGTAACAGCCATTC	AGTGCGTTCGTAGTTTGAGTTTGC
ND5	ATCGGCTGAGAGGGCGTAGG	GCTTGAATGGCTGCTGTGTTGG
ND6	TGCTGTGGGTGAAAGAGTATG	CCCATAATCATACAAAGCCCC
CYTB	GCGTCCTTGCCCTATTACTATCC	GCTTACTGGTTGTCCTCCGATTC
COXI	GCCATAACCCAATACCAAACG	TTGAGGTTGCGGTCTGTTAG
COXII	CTAGTCCTGTATGCCCTTTTCC	GTAAAGGATGCGTAGGGATGG
COXIII	CCTTTTACCACTCCAGCCTAG	CTCCTGATGCGAGTAATACGG
ATP6	GTGTGGTCGGGTGTGTTATTATTC	CAATCCTACCTCCATCGCTAACC
12sRNA	AACCTCACCACCTCTTGCTCAG	ATGGGCTACACCTTGACCTAACG
16sRNA	CGTGAAGAGGCGGGCATAAC	TGCTCGGAGGTTGGGTTCTG
TARDBP	GGGTAACCGAAGATGAGAACG	CTGGGCTGTAACCGTGGAG
LBP	TCTATGGGTCACCTAGAAGAGC	TCCACTGGGTGTGAAATCCTC
ACKR1	ATGCCGGAGGTGTCATCTAAA	AGAATAACTCTCCTTACGGGTCC
IST1	ACTCCTGCCCAGATGAGAGG	TCACAATCTTCTCCCCGTTCT
GPX3	AGAGCCGGGGACAAGAGAA	ATTTGCCAGCATACTGCTTGA
CCDC3	CCTGGGCTACTTCTCGTGC	GCCCTTGCGAACAGTTTGAAAA
C4B	GGACTTACGTTTGTATCAAGGGC	GCACTCAGTAGTGGTGTTATCCC
